# (*Z*,*Z*)-Selanediylbis(2-propenamides): Novel Class of Organoselenium Compounds with High Glutathione Peroxidase-Like Activity. Regio- and Stereoselective Reaction of Sodium Selenide with 3-Trimethylsilyl-2-propynamides

**DOI:** 10.3390/molecules25245940

**Published:** 2020-12-15

**Authors:** Mikhail V. Andreev, Vladimir A. Potapov, Maxim V. Musalov, Svetlana V. Amosova

**Affiliations:** A. E. Favorsky Irkutsk Institute of Chemistry, Siberian Division of The Russian Academy of Sciences, 1 Favorsky Str., 664033 Irkutsk, Russia; miand@irioch.irk.ru (M.V.A.); musalov_maxim@irioch.irk.ru (M.V.M.); amosova@irioch.irk.ru (S.V.A.)

**Keywords:** (*Z*,*Z*)-3,3′-selanediylbis(2-propenamides), 3-trimethylsilyl-2-propynamides, sodium selenide, glutathione peroxidase-like activity, regioselective reactions, stereoselective reactions, desilylation

## Abstract

The efficient regio- and stereoselective synthesis of (*Z*,*Z*)-3,3′-selanediylbis(2-propenamides) in 76–93% yields was developed based on the reaction of sodium selenide with 3-trimethylsilyl-2-propynamides. (*Z*,*Z*)-3,3′-Selanediylbis(2-propenamides) are a novel class of organoselenium compounds. To date, not a single representative of 3,3′-selanediylbis(2-propenamides) has been described in the literature. Studying glutathione peroxidase-like properties by a model reaction showed that the activity of the obtained products significantly varies depending on the organic moieties in the amide group. Divinyl selenide, which contains two lipophilic cyclohexyl substituents in the amide group, exhibits very high glutathione peroxidase-like activity and this compound is considerably superior to other products in this respect.

## 1. Introduction

Vinyl selenides are important intermediates for organic synthesis [1,2,3,4,5,6,7,8,9]. These compounds have been used for the preparation of a number of valuable products [4,5,6,7,8,9]. Vinyl selenides have found application in the synthesis of functionalized ketones, (*Z*)-allyl alcohols, and unsaturated aldehydes [4]. The cross-coupling of vinyl selenides with terminal alkynes in the presence of a nickel/CuI catalyst at room temperature proceeded with of retention of stereochemical configuration leading to (*Z*)- and (*E*)-enyne derivatives in good yields [5]. The coupling reaction of vinyl selenides with Grignard reagents provided corresponding functionalized alkenes [6,7]. The synthesis of resveratrol and its methoxylated analogues—well known compounds due to their anti-inflammatory, anticancer, antibacterial, and neuroprotective activity, has been proposed based on vinyl selenides [8]. An efficient method for preparation of α-phenylselanyl lactones has been developed from α-(phenylseleno)vinyl tozylates [9].

Some vinyl selenides exhibit antioxidant [10], antinociceptive [11], and hepatoprotective activity [12].

The main methods for the preparation of vinyl selenides include a transition metal catalyzed coupling of vinyl halides with diselenides or selenols [13,14,15,16], reactions of thiols or chalcogenolates with selenoalkynes [14,17,18], and addition of selenium-centered nucleophiles to acetylenes [8,14,19,20,21,22,23].

One of the most useful and atom-economic methods is based on addition reactions of selenoles or selenolate anions with acetylenes [8,14,19,20,21,22]. Examples of these reactions refer mainly to vinyl selenides containing aliphatic or aromatic substituents at the β-carbon atom of the double bond. Examples of the synthesis of vinyl selenides bearing electron-withdrawing groups are scarce in the literature. The synthesis of (*Z,Z*)-bis(2-acylvinyl) selenides by the addition reaction of sodium selenide with organyl ethynyl ketones was developed [23]. 

There are only a few representatives of vinyl selenides containing the amide group [24,25,26,27]. The Pd-catalyzed four-component reaction between sulfonamide, alkyne, diphenyl diselenide, and carbon monoxide afforded substituted 3-(phenylselanyl)propenamides in 65–90% yields [24]. Functionalized 3-(phenylselanyl)propenamides were obtained in 57–89% yields based on 3-(phenylselanyl)acrylic acid which was synthesized in 65% yield from diphenyl diselenide and ethyl propiolate [25]. The reaction of carbamoselenoate, PhSeC(O)NMe_2_, with 1-octyne in the presence of Pd(PPh_3_)_4_ gave 3-hexyl-3-(phenylselanyl)propenamide in 40% yield [26].

**Figure 1:**
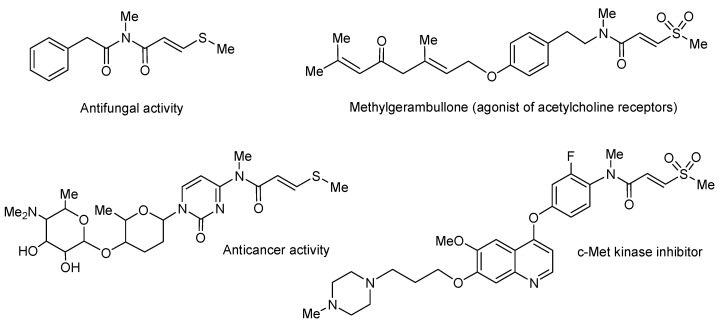
Known biologically active derivatives of vinyl sulfides containing the amide group (anticancer [28], antifungal [29], agonist of acetylcholine receptors [30], c-Met kinase inhibitor [31]).

There are no data in the literature about the biological activity of 3-selanylpropenamides. However, it is known that vinyl sulfides bearing the amide group in the β-position exhibit anticancer [28] and antifungal [29] activity (Figure 1). Containing the 2-amidovinylsulfonyl group methylgerambullone (isolated from *Glycosmis angustifolia*) acts as the agonist of acetylcholine receptors [30]. Phenoxyquinolines bearing a 2-amidovinylsulfonyl moiety shows the properties of c-Met kinase inhibitors [31] (Figure 1). Taking into account the indicated biological properties of 3-sulfanylpropenamides, it can be assumed that selenium analogs of these compounds can also display some kinds of biological activity. Moreover, the vinylamide group, itself, is an important part of some biologically active natural compounds and pharmaceuticals, which exhibit antitumor, anti-tuberculosis, and anticonvulsant activity [32,33,34,35,36]. 

**Figure 2:**
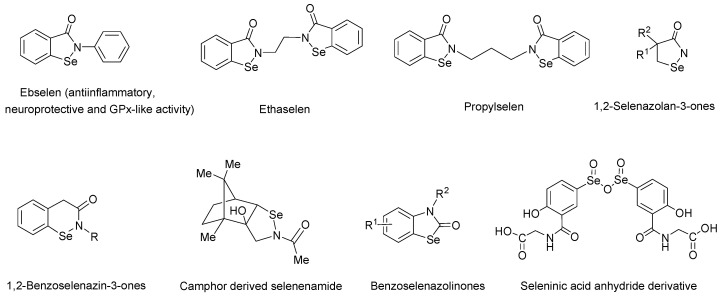
Containing amide group organoselenium compounds, which exhibit glutathione peroxidase-like activity.

To date, considerable effort has been devoted to the discovery of compounds that mimic the action of selenium-containing glutathione peroxidase enzymes [37,38,39,40,41,42,43,44,45,46,47]. The presence of selenium in these enzymes largely determines the glutathione peroxidase activity. In particular, organoselenium compounds bearing amide groups have been shown to be good catalysts for the reduction of peroxides and hydroperoxides with thiols (Figure 2). 

Ebselen, which contains the selenenamide function in the cycle, and its analog ethaselen and propylselen show high glutathione peroxidase mimetic properties [37,38,39,40,41]. Additionally, ebselen exhibits anti-inflammatory and neuroprotective activity. These properties combined with glutathione peroxidase-like activity and relatively low toxicity of ebselen has led to therapeutic application of this compound, which has undergone evaluation in clinical trials as an anti-inflammatory agent [42]. This compound is also used for the treatment and prevention of cardiovascular diseases and ischemic stroke [41]. 

The range of bearing amide group organoselenium compounds, which exhibit glutathione peroxidase activity, comprises 1,2-benzoselenazin-3-ones (including the homologue of ebselen, 2-phenyl-1,2-benzoselenazin-3-one), 1,2-selenazolan-3-ones, benzoselenazolinones, and seleninic acid anhydride (which has been synthesized based on salicylic acid amide and selenium tetrachloride) [41,42,43,44] (Figure 2). 

Camphor-derived selenenamide was synthesized by action of bromine on corresponding camphor diselenide, which was obtained based on the reaction of camphor enolate with selenium [42]. The glutathione peroxidase mimetic property of the camphor derived selenenamide was studied using a model reaction of benzenemethanethiol oxidation by *tert*-butyl hydroperoxide in the presence of the selenenamide as a catalyst (10% mol) in dichloromethane or deuterochloroform at room temperature. A similar model reaction of benzenemethanethiol oxidation by hydrogen peroxide was applied to examine the glutathione peroxidase-like activity of containing hydroxy group divinyl selenides as a catalysts (10% mol) [45]. The progress of the reaction was monitored by ^1^H NMR spectroscopy. 

Previously we developed efficient syntheses of vinyl and divinyl selenides based on the addition of selenium-containing reagents, including sodium selenide, to the triple bond of acetylene and its derivatives [23,27,48,49,50,51,52,53,54,55,56,57].

To date, the reactions of sodium selenide with neither 2-propynamides nor 3-(triorganylsilyl)-2-propynamides have yet been described in the literature. It is known that the introduction of electron-donating triorganylsilyl group at the triple bond changes the reactivity of acetylene derivatives and deactivates the triple bond toward nucleophilic addition [58].

In order to develop the method for preparation of previously unknown divinyl selenides containing amide groups we studied the reaction of sodium selenide with 3-(trimethylsilyl)-2-propynamides and found the conditions for regio- and stereoselective addition. The obtained results are described in the present work.

## 2. Results and Discussion

**Scheme 1:**
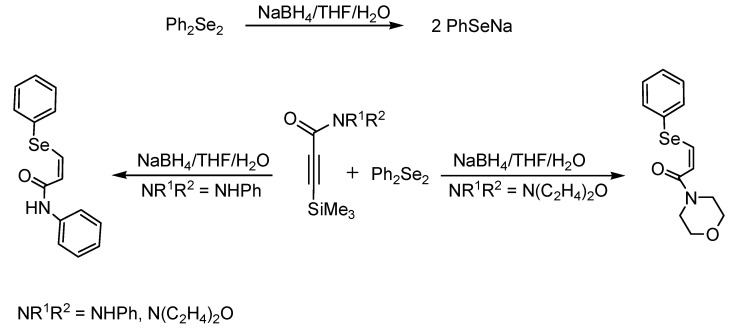
Synthesis of containing the amide group vinyl selenides from diphenyl diselenide and 3-(trimethylsilyl)-2-propynamides.

Recently we realized the addition of sodium benzeneselenolate to 3-(trimethylsilyl)-2-propynamides containing morpholine and phenylamide moieties (Scheme 1) [27]. The reaction was carried out by addition of sodium borohydride to a stirred solution of 3-trimethylsilyl-2-propynamides and diphenyl diselenide in a THF–water (4/1) system at room temperature and accompanied by desilylation. The generation of sodium benzeneselenolate occurred in situ followed by nucleophilic addition of this highly reactive intermediate to the triple bond. 

The reaction proceeded in stereo- and regioselective manners affording (*Z*)-*N*-phenyl-3-(phenylselanyl)prop-2-enamide (72% yield) and (*Z*)-1-morpholino-3-(phenylselanyl)prop-2-en-1-one (70% yield), which were isolated as colorless crystalline compounds [27]. To the best of our knowledge, these are first examples of the addition of organylselenolates to 3-silyl-2-propynamides. 

The commonly used conditions for generation of organylselenolates from corresponding diselenides and sodium selenide from elemental selenium consist in the application of sodium borohydride as a reducing agent and carrying out the reaction in alcohols [59]. However, when the reactions of sodium benzeneselenolate or sodium selenide with 3-(trimethylsilyl)-2-propynamides proceeded in methanol or ethanol, the formation of 3-alkoxy-2-propenamides as by-products was observed. The possibility of the formation of 3-alkoxy-2-propenamides from 3-(trimethylsilyl)-2-propynamides in reactions with alcohols has been previously described [60].

We found that the THF–water system is preferable in addition reactions of selenium-centered nucleophiles with 3-(trimethylsilyl)-2-propynamides compared to commonly used alcohol conditions. The yields of the target products are higher and 3-alkoxy-2-propenamides are not formed as by-products. 

**Scheme 2:**
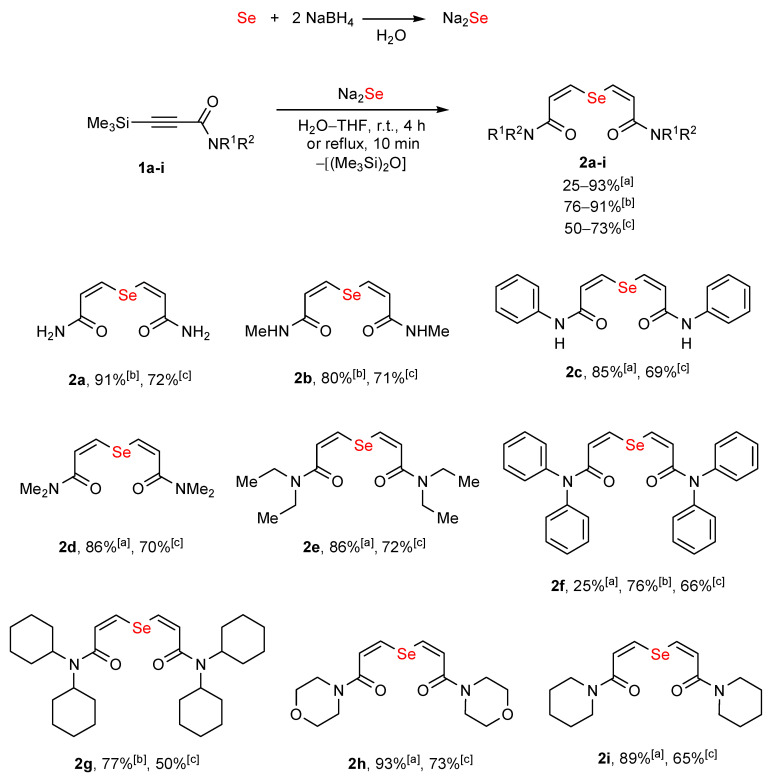
Synthesis of divinyl selenides **2a**–**i** from 3-(trimethylsilyl)-2-propynamides **1a**–**i**, elemental selenium, and sodium borohydride. Sodium selenide was obtained by addition of an aqueous solution of sodium borohydride to a hot mixture of selenium and water (Methods **A** and **B**). ^[a]^ Method **A**: THF/water~1:3. A solution of 3-(trimethylsilyl)-2-propynamide in THF was added to a hot aqueous solution of sodium selenide and the mixture was refluxed for 10 min. Yields: 25–93%. ^[b]^ Method **B**: THF/water~3:1. A solution of 3-(trimethylsilyl)-2-propynamide in THF was added to a hot aqueous solution of sodium selenide and the mixture was refluxed for 10 min. Yields: 76–91%. ^[c]^ Method **C**: sodium borohydride was added portionwise to a mixture of 3-(trimethylsilyl)-2-propynamide, selenium, THF and water and the mixture was stirred at room temperature for 4 h (10 h for **2g**). Yields: 50–73%.

The addition reactions of selenide anion with propynamides and 3-silyl-2-propynamides have not yet been described in the literature. In order to obtain previously unknown divinyl selenides containing amide groups we studied the addition of sodium selenide to 3-(trimethylsilyl)-2-propynamides bearing various groups (phenyl, alkyl, cyclohexyl, morpholine and piperidine) in the amide moieties (Scheme 2). Sodium selenide was efficiently generated from elemental selenium and sodium borohydride in water and used without isolation in further nucleophilic addition reactions. 

The conditions for the regio- and stereoselective reaction of 3-trimethylsilyl-2-propynamides **1a–i** with sodium selenide were found. The reaction proceeded in the THF–water system under argon affording (*Z,Z*)-3,3′-selanediylbis(2-propynamides) **2a**–**i** in 76-93% yields (Scheme 2). 

Water is necessary for generating sodium selenide and reacting Na_2_Se with propynamides **1a**–**i** which are soluble in THF. The ratio of the solvents in the THF–water system was varied from 1/3 (method **A**) to 3/1(method **B**). In the method **A** and **B**, a solution of silylpropynamides in THF was added to a hot aqueous solution of sodium selenide, which was obtained from elemental selenium and sodium borohydride, and the mixture was refluxed for 10 min. In the case of method **C**, sodium borohydride was added portionwise to a mixture of propynamides **1b**–**d**,**f**–**i** and selenium in the THF–water system (the ratio of the solvents 4/1) and the mixture was stirred at room temperature for 4 h (10 h for **2g**). 

Best yields of the products were obtained when the reaction mixtures were refluxed for 10 min in the THF–water system. When reaction was carried out at room temperature, the yields dropped despite increasing the reaction duration. The divinyl selenides **2b**–**d**,**f**–**i** were obtained in 50–73% yields with 4 h stirring at room temperature under argon (Scheme 2, method **C**). Surprisingly, neither unconverted 3-(trimethylsilyl)-2-propynamides **1b**–**d**,**f**–**i** nor desilylated propynamides were detected in the reaction mixture in these cases after completion of the reaction (method **C**). However, the yields of the target products **2b**–**d**,**f**–**i** were lower than in the methods **A** and **B**.

The reaction of sodium selenide with propynamide **1f** bearing two phenyl substituents in the amide group under the conditions of method **A** led to a mixture containing divinyl selenide **2f** in 25% yield, unconverted silylpropynamide **1f** (31% conversion) and the desilylated amide, *N*,*N*-diphenyl-2-propynamide (1%). The reaction of sodium selenide with silylpropynamide **1g** containing two cyclohexyl moieties in the amide group also gave similar poor results. We supposed that the reason of the insufficient yield of selenides **2f**,**g** and low conversion of starting amides **1f**,**g** may be poor solubility of propyneamides **1f**,**g** in the mixture water–THF (3/1, method **A**) due to lipophilic organic moieties of the amide group. The silylpropynamides **1f**,**g** are insoluble in water but soluble in THF. Indeed, when the method **B** (THF–water 3/1) was applied, products **2f**,**g** were obtained in 76–77% yields. The method **A** was found to provide high yields of products **2c**–**e**,**h**,**i** (85–93%) derived from silylpropynamides **1c**–**e**,**h**,**i** containing monophenyl, dialkyl, morpholine, and piperidine moieties in the amide group.

**Scheme 3:**
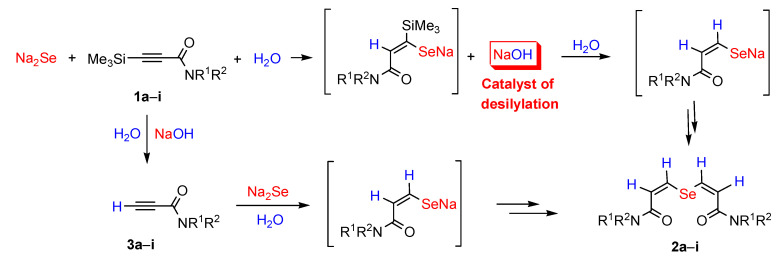
The possible reaction pathways for the formation of products **2a**–**i**.

The possible pathway for the formation of products **2a**–**i** can include both the addition—desilylation processes and the sequential desilylation—addition reactions via the generation of intermediate propynamides **3a**–**i** (Scheme 3). The addition of sodium selenide to the triple bond of silylpropynamides **1a**–**i** is accompanied by the formation of sodium hydroxide, which acts as the catalyst for the desilylation reaction. We suppose that the desilylation process can proceed on different stages of the reaction including various intermediate species (Scheme 3). 

The formation of intermediate 2-propynamides **3a**–**i** in very small amounts (before the isolation of the reaction products **2a**–**i**) was registered in the reaction mixture by NMR. The NMR data of the intermediate propynamides **3a**–**i** coincide with the spectral characteristics of the previously obtained samples of these compounds, which were synthesized by desilylation of silylpropynamides **1a**–**i** [60].

The formation of propynamides **3a**–**i** in the reaction (Scheme 2) indicates the possibility of the reaction pathway via desilylation of silylpropynamides **1a**–**i**. It was previously established that silylpropynamides **1a**–**i** can be desilylated by the action of various reagents (potassium fluoride, alkali metal hydroxides and other bases) and converted to corresponding propynamides **3a**–**i** [60].

**Scheme 4:**
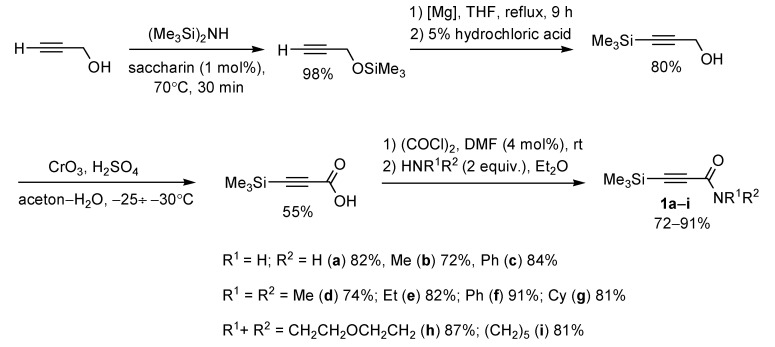
The method for the preparation of 3-trimethylsilyl-2-propynamides **1a**–**i**.

It is worth noting that the application of 3-trimethylsilyl-2-propynamides **1a**–**i** as the initial substrates in the preparation of the target vinyl selenides is preferable compared to 2-propynamides with the terminal triple bond. The latter compounds are hardly available and the price for these chemicals is very high. Their preparation is usually based on toxic and skin-irritating propynoic acid. The silylpropynamides **1a**–**i** were synthesized in the present work by the method depicted in Scheme 4 [61,62,63]. Inexpensive starting propargyl alcohol, good selectivity of these reactions and high yield of the target products allowed to make silylpropynamides **1a**–**i** readily available compounds and to use them in the synthesis of valuable products [64,65,66].

The obtained selanediylbis(2-propynamides) **2a**–**i** are a novel class of organoselenium compounds. Like ebselen and some organoselenium compounds, which exhibit glutathione peroxidase-like activity (Figure 2), products **2a**–**i** contain the amide function, and their activity deserved to be studied.

We studied glutathione peroxidase-like activity of the obtained products **2a**–**i** using the model reaction of benzenemethanethiol oxidation [42,45] by *tert*-butyl hydroperoxide (TBHP) in the presence of compounds **2a**–**i** as catalysts and the progress of this reaction was monitored by ^1^H NMR spectroscopy. First experiments in the NMR tubes (TBHP, BnSH, 0.1 mmol, deuterochloroform) at room temperature showed that the reactions proceeded too fast when 10% mol of the catalysts were used. In order to realize the ^1^H NMR monitoring, the amounts of the catalysts were decreased to 0.5% mol. Diphenyl diselenide was used as the standard compound (this compound is often used as the standard catalyst for in these experiments [42,43,44,45,46,47]).

**Figure 3:**
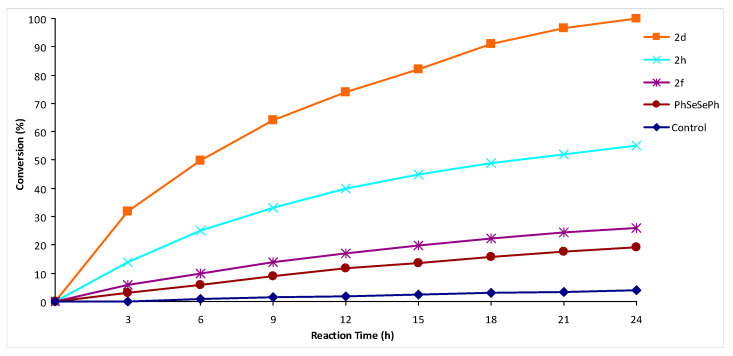
Studying glutathione peroxidase-like activity of compounds **2d**,**f**,**h** by ^1^H NMR monitoring with the use of Ph_2_Se_2_ as the standard compound (TBHP, BnSH, 0.1 mmol, deuterochloroform, 0.5% mol of studied compounds). The control experiment was carried out in the absence of studied compounds.

**Figure 4:**
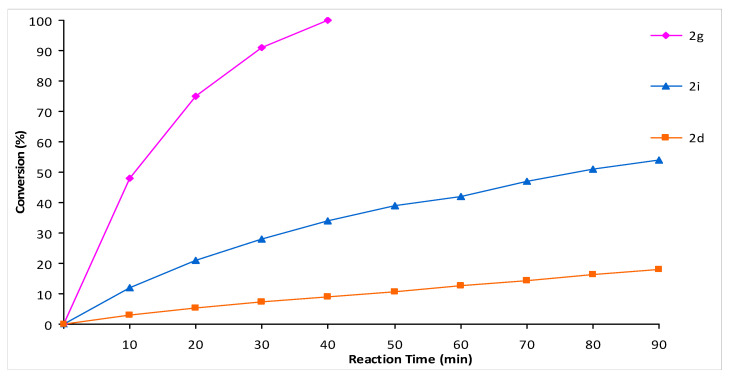
Studying glutathione peroxidase-like activity of compounds **2d**,**g**,**i** (TBHP, BnSH, 0.1 mmol, deuterochloroform, 0.5% mol of studied compounds) by ^1^H NMR monitoring.

It was found that the activity of the obtained products **2a**–**i** varies significantly depending on the organic moieties in the amide group. The results of studying the compounds **2d**,**f**,**g**,**h**,**i**, which outperform diphenyl diselenide in the glutathione peroxidase-like activity, are presented in Figure 3 (a 24 h scale) and Figure 4 (a 90 min scale). In the control experiment, under the same reaction conditions but in the absence of the catalyst, the conversion of phenylmethanethiol was only about 4% after 24 h according to ^1^H NMR data.

**Figure 5:**
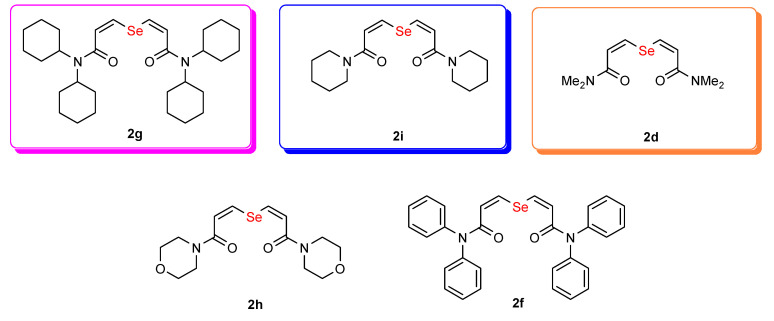
Compounds **2d**,**f**,**g**,**h**,**i** exhibiting higher glutathione peroxidase-like activity compared to Ph_2_Se_2_ (the compounds are arranged in the decreasing order of the activity).

Product **2g** containing two lipophilic cyclohexyl substituents in the amide group shows highest glutathione peroxidase-like properties (Figure 4 and Figure 5). This compound is significantly superior to other products in activity. The second most active product is compound **2i** (Figure 4) bearing the piperidine moieties in the amide function and the third is product **2d** (the activity of which is presented in both Figure 3 and Figure 4). Compounds **2f**,**h** containing the morpholine and phenyl moieties also exhibit higher activity compared to diphenyl diselenide. 

The obtained results are very promising. However, the interpretation of the influence of organic moieties on the catalytic activity and discussion on possible intermediates of the catalytic cycle requires additional data and further research. 

## 3. Experimental Section

### 3.1. General Information

The ^1^H (400.1 MHz), ^13^C (100.6 MHz) and ^77^Se (76.3 MHz) NMR spectra (Supplementary Materials) were recorded on a Bruker DPX-400 spectrometer (Bruker BioSpin GmbH, Rheinstetten, Germany) in CDCl_3_ and *d*_6_-DMSO 5–10% solutions and referred to TMS (^1^H, ^13^C) and dimethyl selenide (^77^Se). The IR spectra were taken on a Bruker IFS-25 spectrometer (Bruker BioSpin GmbH, Rheinstetten, Germany). Mass spectra were recorded on a Shimadzu GCMS-QP5050A (Shimadzu Corporation, Kyoto, Japan) with electron impact (EI) ionization (70 eV). Elemental analysis was performed on a Thermo Scientific Flash 2000 Elemental Analyzer (Thermo Fisher Scientific Inc., Milan, Italy). Melting points were determined on a Kofler Hot-Stage Microscope PolyTherm A apparatus (Wagner & Munz GmbH, München, Germany). The organic solvents were dried and distilled according to standard procedures. Silica gel (Alfa Aesar, 0.06–0.20 mm (70–230 mesh)) and ethyl acetat–methanol (10:1) as an eluent were used for column chromatography. 

### 3.2. Method ***A*** (Preparation of Compounds ***2c**–**f**,**h**,**i***)

A mixture of elemental selenium (19 mg, 0.24 mmol) and degassed water (4.0 mL) was heated on a water bath (90–95 °C) and a solution of NaBH_4_ (40 mg, 1.05 mmol) in degassed water (0.5 mL) was added under argon. After dissolution of selenium and the formation of colorless mixture, a solution of 3-trimethylsilyl-2-propynamide (0.48 mmol) in THF (1.5 mL) was added to a hot aqueous solution of the sodium selenide and the mixture was refluxed for 10 min (5 h for **1g**) under argon. The mixture was cooled by cold water bath and extracted with CH_2_Cl_2_ (3 × 7.0 mL). The organic phase was dried over Na_2_SO_4_ and the solvent was removed under reduced pressure. General yields: 85–93%.

### 3.3. Method ***B*** (Preparation of Compounds ***2a**,**b**,**f**,**g***)

A mixture of elemental selenium (19 mg, 0.24 mmol) and degassed water (2.0 mL) was heated on a water bath (90–95 °C) and a solution of NaBH_4_ (40 mg, 1.05 mmol) in degassed water (0.4 mL) was added under argon. After dissolution of selenium and the formation of colorless mixture, a solution of 3-trimethylsilyl-2-propynamide (0.48 mmol) in THF (7.0 mL) was added to a hot aqueous solution of the sodium selenide and the mixture was refluxed for 10 min (5 h for **1g**) under argon. The mixture was cooled by cold water bath and THF was removed by a rotary evaporator. The residue was extracted with CH_2_Cl_2_ (3 × 7.0 mL). The organic phase was dried over Na_2_SO_4_ and the solvent was removed under reduced pressure. General yields: 76–91%.

### 3.4. Method ***C*** (Preparation of Compounds ***2a**–**i***)

NaBH_4_ (30 mg, 0.79 mmol) was added portionwise to a stirred mixture of selenium (19 mg, 0.24 mmol), 3-trimethylsilyl-2-propynamide (0.48 mmol), degassed water (0.5 mL) and THF (2.0 mL). The mixture was stirred at room temperature for 5 h under argon and degassed water (2 mL) was added. The mixture was extracted with CH_2_Cl_2_ (3 × 7.0 mL). The organic phase was dried over Na_2_SO_4_ and the solvent was removed under reduced pressure. General yields: 50–74%.

### 3.5. Compounds ***2a**–**i***

*(Z)-3-[(Z)-3-amino-3-oxo-1-propenyl]selanyl-2-propenamide* (**2a**) was prepared by the method B (91% yield) and the method **C** (72% yield). After refluxing for 10 min, the solvent was removed under reduced pressure and product **2a** was extracted from the residue by boiling acetone. The solvent was removed under reduced pressure giving **2a** (48 mg, 91%, method **B**); (38 mg, 72%, method **C**); yellowish powder; mp 183–184 °C. ^1^H NMR (400 MHz, *d*_6_-DMSO): δ 6.38 (d, ^3^*J* = 9.5 Hz, 2H, =CHCO), 7.10 (s, 2H, NH_2_), 7.50 (s, 2H, NH_2_), 7.65 (d, ^3^*J* = 9.5 Hz, 2H, SeCH=). ^13^C NMR (100 MHz, *d*_6_-DMSO): δ 120.6 (=CCO), 145.3 (SeC=, ^1^*J*_Se–C_ = 128.2 Hz), 168.1 (C=O). ^77^Se NMR (76 MHz, *d*_6_-DMSO): δ 507.4. IR (KBr): 3430, 3389, 3180, 2929, 1654 (C=O), 1575 (C=C), 1558 (C=C), 1397, 1282, 1162, 1111, 1050, 928, 802, 729, 632, 589, 524 cm^–1^. MS (EI), *m*/*z* (%): 220 [M]^+^, 218 (13), 152 (4), 150 (24), 148 (12), 134 (6), 133 (9), 106 (9), 88 (100), 71 (9), 70 (22), 55 (6), 44 (54), 43 (6). Anal. calcd for C_6_H_8_N_2_O_2_Se (219.10): C 32.89, H 3.68, N 12.79, Se 36.04%. Found: C 32.73, H 3.57, N 12.93, Se 35.89%.

*(Z)-N-methyl-3-[(Z)-3-(methylamino)-3-oxo-1-propenyl]selanyl-2-propenamide* (**2b**) was prepared by the method **B** (80% yield) and the method **C** (71% yield). After addition of degassed water to the reaction mixture, the precipitate was filtered and dried in vacuum giving product **2b** (47.5 mg, 80%, method **B**); (42 mg, 71%, method **C**); white powder; mp 215–216 °C. ^1^H NMR (400 MHz, *d*_6_-DMSO): δ 2.64 (d, ^3^*J* = 4.4 Hz, 6H, CH_3_), 6.35 (d, ^3^*J* = 9.6 Hz, 2H, =CHCO), 7.59 (d, ^3^*J* = 9.6 Hz, 2H, SeCH=), 8.01 (q, ^3^*J* = 4.4 Hz, 2H, NH). ^13^C NMR (100 MHz, *d*_6_-DMSO): δ 25.5 (CH_3_), 120.4 (=CCO), 144.0 (SeC=, ^1^*J*_Se–C_ = 127.4 Hz), 166.8 (C=O). ^77^Se NMR (76 MHz, *d*_6_-DMSO): δ 502.7. IR (KBr): 3324, 3041, 2938, 1644 (C=O), 1580 (C=C), 1524 (C=C), 1413, 1240, 1184, 1057, 805, 725, 698, 653 cm^–1^. MS (EI), *m*/*z* (%): 248 (20) [M]^+^, 246 (9), 187 (6), 166 (9), 164 (43), 162 (30), 161 (25), 147 (92), 145 (45), 143 (22), 135 (9), 134 (15), 133 (10), 132 (7), 131 (7), 110 (11), 107 (13), 106 (15), 105 (8), 104 (9), 84 (100), 68 (17), 66 (7), 58 (96), 57 (7), 56 (15), 55 (15), 53 (13), 44 (14), 43 (9), 42 (19), 41 (10). Anal. calcd for C_8_H_12_N_2_O_2_Se (247.15): C 38.88, H 4.89, N 11.33, Se 31.95%. Found: C 38.91, H 4.86, N 11.57, Se 32.14%. 

*(Z)-N-phenyl-3-[(Z)-3-anilino-3-oxo-1-propenyl]selanyl-2-propenamide* (**2c**) was prepared by the method **A** (85% yield) and the method **C** (69% yield). After removing the solvent, the residue was dissolved in THF and precipitated with cold hexane giving product **2c** which was dried in vacuum (76 mg, 85%, method **A**); (61 mg, 69%, method **C**); beige powder; mp 219–220 °C. ^1^H NMR (400 MHz, *d*_6_-DMSO): δ 6.65 (d, ^3^*J* = 9.6 Hz, 2H, =CHCO), 7.06 (t, ^3^*J* = 7.7 Hz, 2H, H*^p^*), 7.32 (dd, ^3^*J* = 7.7 Hz, 4H, H*^m^*), 7.66 (d, ^3^*J* = 7.7 Hz, 4H, H*^o^*), 7.94 (d, ^3^*J* = 9.6 Hz, 2H, SeCH=), 10.23 (s, 2H, NH). ^13^C NMR (100 MHz, *d*_6_-DMSO): δ 119.0 (=CCO), 121.0 (C*^o^*), 123.4 (C*^p^*), 128.9 (C*^m^*), 139.1 (C*^i^*), 146.7 (SeC=, ^1^*J*_Se–C_ = 129.0 Hz), 164.9 (C=O). ^77^Se NMR (76 MHz, *d*_6_-DMSO): δ 518.8. IR (KBr): 3266, 3129, 3039, 2929, 1636 (C=O), 1603 (C=C, Ph), 1544 (C=C, Ph), 1499 (C=C, Ph), 1439, 1365, 1302, 1247, 1201, 1159, 979, 796, 744, 689, 594, 505 cm^–1^. MS (EI), *m*/*z* (%): 372 (8) [M]^+^, 224 (15), 211 (9), 209 (47), 207 (23), 206 (9), 205 (9), 187 (6), 161 (18), 159 (11), 147 (13), 146 (100), 145 (12), 133 (8), 132 (31), 131 (6), 128 (9), 120 (8), 117 (14), 106 (8), 104 (12), 94 (11), 93 (79), 92 (29), 91 (9), 77 (37), 66 (11), 65 (34), 64 (7), 51 (11), 39 (17). Anal. calcd for C_18_H_16_N_2_O_2_Se (371.29): C 58.23, H 4.34, N 7.54, Se 21.27%. Found: C 58.50, H 4.29, N 7.48, 20.97%. 

*(Z)-3-[(Z)-3-(dimethylamino)-3-oxo-1-propenyl]selanyl-N,N-dimethyl-2-propenamide* (**2d**) was prepared by the method **A** (86% yield) and the method **C** (68% yield). After removing the solvent, the residue was dissolved in CHCl_3_ and precipitated with cold hexane giving product **2d**, which was dried in vacuum (57 mg, 86%, method **A**); (46 mg, 68%, method **C**); white powder; mp 181–182 °C. ^1^H NMR (400 MHz, CDCl_3_): δ 3.01, 3.06 (s, 12H, CH_3_), 6.74 (d, ^3^*J* = 9.7 Hz, 2H, =CHCO), 7.52 (d, ^3^*J* = 9.7 Hz, 2H, SeCH=). ^13^C NMR (100 MHz, CDCl_3_): δ 35.4, 37.2 (CH_3_), 116.8 (=CCO), 147.6 (SeC=, ^1^*J*_Se–C_ = 132.6 Hz), 166.9 (C=O). ^77^Se NMR (76 MHz, CDCl_3_): δ 516.8. IR (KBr): 3024, 2925, 2861, 1624 (C=O), 1572 (C=C), 1492, 1403, 1324, 1261, 1146, 1065, 974, 868, 791, 649, 591, 523 cm^−1^. MS (EI), *m/z* (%): 276 (8) [M]^+^, 195 (10), 187 (8), 178 (25), 176 (12), 161 (7), 124 (8), 106 (6), 98 (71), 72 (100), 70 (9), 55 (9), 46 (12), 44 (29), 15 (42). Anal. calcd for C_10_H_16_N_2_O_2_Se (275.21): C 43.64, H 5.86, N 10.18, Se 28.69%. Found: C 43.71, H 5.63, N 10.29, Se 28.54%. 

*(Z)-3-[(Z)-3-(diethylamino)-3-oxo-1-propenyl]selanyl-N,N-diethyl-2-propenamide* (**2e**) was prepared by the method **A** (86% yield) and the method **C** (72% yield). After removing the solvent, residue was dissolved in CHCl_3_ and precipitated with cold hexane in a refrigerator (−18 °C) giving product **2e** which was dried in vacuum (68 mg, 86%, method **A**); (57 mg, 72%, method **C**); pale yellow solid; mp 56–57 °C. ^1^H NMR (400 MHz, CDCl_3_): δ 1.12 (t, ^3^*J* = 6.8 Hz, 12H, CH_3_), 3.32, 3.38 (q, ^3^*J* = 6.8 Hz, 8H, CH_2_), 6.64 (d, ^3^*J* = 9.7 Hz, 2H, =CHCO), 7.47 (d, ^3^*J* = 9.7 Hz, 2H, SeCH=). ^13^C NMR (100 MHz, CDCl_3_): δ 13.2, 14.8 (CH_3_), 40.6, 42.1 (CH_2_), 117.1 (=CCO), 147.5 (SeC=, ^1^*J*_Se–C_ = 131.9 Hz), 166 (C=O). ^77^Se NMR (76 MHz, CDCl_3_): δ 519.4. IR (KBr): 3027, 2975, 2930, 2901, 2873, 1618 (C=O), 1566 (C=C), 1481, 1448, 1428, 1376, 1360, 1304, 1258, 1218, 1141, 1077, 949, 836, 791, 638, 592 cm^−1^. Anal. calcd for C_14_H_24_N_2_O_2_Se (331.31): C 50.75, H 7.30, N 8.46, Se 23.83%. Found: C 50.56, H 7.10, N 8.31, Se 23.72%.

*(Z)-3-[(Z)-3-(diphenylamino)-3-oxo-1-propenyl]selanyl-N,N-diphenyl-2-propenamide* (**2f**) was prepared by the method **A** (25% yield), the method **B** (76% yield) and the method **C** (66% yield). After removing the solvent, the residue was dissolved in CHCl_3_ and precipitated with cold hexane giving product **2f** which was dried in vacuum (31 mg, 25%, method **A**); (96 mg, 76%, method **B**); (83 mg, 66%, method **C**); beige powder; mp 201–203 °C. ^1^H NMR (400 MHz, CDCl_3_): δ 6.35 (d, ^3^*J* = 9.7 Hz, 2H, =CHCO), 7.23–7.30 (m, 12H, H*^o,p^*), 7.32–7.40 (m, 8H, H*^m^*), 7.48 (d, ^3^*J* = 9.7 Hz, 2H, SeCH=). ^13^C NMR (100 MHz, CDCl_3_): δ 119.6 (=CCO), 125.1–128.6 (C*^o^*^,*p*^), 129.2 (C*^m^*), 142.6 (C*^i^*), 149.2 (SeC=, ^1^*J*_Se–C_ = 133.9 Hz), 166.3 (C=O). ^77^Se NMR (76 MHz, CDCl_3_): δ 533.3. IR (KBr): 2929, 1635 (C=O), 1552 (C=C, Ph), 1492, 1370, 1269, 1164, 1082, 1035, 782, 756, 695, 543 cm^−1^. MS (EI), *m/z* (%): 523 (2) [M]^+^, 303 (6), 222 (14), 209 (20), 208 (100), 196 (9), 180 (29), 170 (9), 169 (61), 168 (38), 167 (44), 166 (6), 77 (19). Anal. calcd for C_30_H_24_N_2_O_2_Se (523.48): C 68.83, H 4.62, N 5.35, Se 15.08%. Found: C 68.65, H 4.47, N 5.56, Se 15.04%. 

*(Z)-3-[(Z)-3-(dicyclohexylamino)-3-oxo-1-propenyl]selanyl-N,N-dicyclohexyl-2-propenamide* (**2g**) was prepared by the method **B** (refluxing for 5 h, 77% yield) and method **C** (stirring at room temperature for 10 h, 50% yield). After removing the solvent, the residue was recrystallized from benzene (101 mg, 77%, method **A**); (66 mg, 50%, method **C**); white powder; mp 127–129 °C. ^1^H NMR (400 MHz, CDCl_3_): δ 1.03–1.38, 1.44–1.90 (m, 36H, H), 2.12–2.27 (m, 4H, H), 3.37–3.54 (m, 4H, H*^1^*), 6.70 (d, ^3^*J* = 9.7 Hz, 2H, =CHCO), 7.39 (d, ^3^*J* = 9.7 Hz, 2H, SeCH=). ^13^C NMR (100 MHz, CDCl_3_): δ 24.4 (C*^3^*^,*4*,*5*^), 25.4 (C*^3^*^,*5*^), 29.4, 30.6, 31.0 (C*^2^*^,*6*^), 54.5, 56.0 (C*^1^*), 118.8 (=CCO), 145.0 (SeC=, ^1^*J*_Se–C_ = 130.6 Hz), 165.4 (C=O). ^77^Se NMR (76 MHz, CDCl_3_): δ 508.7. IR (KBr): 2926, 2852, 2663, 1614 (C=O), 1559 (C=C), 1465, 1439, 1389, 1366, 1342, 1291, 1264, 1233, 1181, 1141, 1126, 1053, 996, 896, 779, 714, 637, 619, 595, 504. MS (EI), *m*/*z* (%): 549 (4) [M]^+^, 314 (20), 312 (10), 286 (6), 235 (19), 234 (89), 232 (9), 181 (8), 180 (44), 178 (6), 161 (10), 152 (47), 150 (19), 148 (7), 138 (19), 98 (69), 96 (11), 83 (49), 82 (9), 81 (18), 79 (8), 70 (12), 67 (9), 56 (21), 55 (100), 44 (10), 43 (8), 41 (42). Anal. calcd for C_30_H_48_N_2_O_2_Se (547.67): C 65.79, H 8.83, N 5.11, Se 14.42%. Found: C 65.63, H 8.82, N 4.96, Se 14.38%.

*(Z)-1-morpholino-3-[(Z)-3-morpholino-3-oxo-1-propenyl]selanyl-2-propen-1-one* (**2h**) was prepared by the method **A** (93% yield) and the method **C** (73% yield). After removing the solvent, residue was dissolved in CHCl_3_ and precipitated with cold hexane (80 mg, 93%, method **A**); (63 mg, 73%, method **C**); white powder; mp 196–197 °C. ^1^H NMR (400 MHz, CDCl_3_): δ 3.55 (br m, 4H, H*^3^*^,*5*^), 3.69 (br m, 12H, H*^3^*^,*5*^, H*^2^*^,*6*^), 6.72 (d, ^3^*J* = 9.7 Hz, 2H, =CHCO), 7.59 (d, ^3^*J* = 9.7 Hz, 2H, SeCH=). ^13^C NMR (100.6 MHz, CDCl_3_): δ 42.1, 46.0 (C*^3^*^,*5*^), 66.8 (C*^2^*^,*6*^), 116.0 (=CCO), 148.7 (SeC=, ^1^*J*_Se–C_ = 132.2 Hz), 165.7 (C=O). ^77^Se NMR (76 MHz, CDCl_3_): δ 520.2. IR (KBr): 2957, 2910, 1616 (C=O), 1563 (C=C), 1437, 1235, 1112, 1035, 964, 786, 602, 572 cm^−1^. Anal. calcd for C_14_H_20_N_2_O_4_Se (359.28): C 46.80, H 5.61, N 7.80, Se 21.98%. Found: C 46.96, H 5.39, 7.95, Se 21.60%. MS (EI), *m/z* (%): 549 (4) [M]^+^, 279 (11), 220 (27), 218 (13), 187 (14), 166 (11), 159 (15), 161 (26), 159 (14), 141 (11), 140 (100), 135 (9), 134 (9), 133 (10), 132 (7), 131 (8), 124 (6), 114 (79), 107 (7), 106 (8), 88 (17), 87 (16), 86 (99), 82 (13), 70 (77), 57 (17), 56 (55), 55 (30), 54 (9), 53 (9), 45 (8), 44 (6), 43 (6), 42 (40), 41 (10).

*(Z)-3-[(Z)-3-piperidino-3-oxo-1-propenyl]selanyl-1-piperidino-2-propen-1-one* (**2i**) was prepared by the method **A** (89% yield) and the method **C** (65% yield). After removing the solvent, residue was dissolved in CHCl_3_ and precipitated with cold hexane (77 mg, 89%, method **A**); (55 mg, 65%, method **C**); beige powder; mp 208–209 °C. ^1^H NMR (400 MHz, CDCl_3_): δ 1.53–1.61 (m, 8H, H*^3^*^,*5*^), 1.61–1.70 (m, 4H, H*^4^*), 3.49, 3.61 (br m, 8H, H*^2^*^,*6*^), 6.75 (d, ^3^*J* = 9.8 Hz, 2H, =CHCO), 7.49 (d, ^3^*J* = 9.8 Hz, 2H, SeCH=). ^13^C NMR (100 MHz, CDCl_3_): δ 24.7 (C*^4^*), 25.6, 26.7 (C*^3^*^,*5*^), 42.9, 46.8 (C*^2^*^,*6*^), 117.0 (=CCO), 147.1 (SeC=, ^1^*J*_Se–C_ = 131.0 Hz), 165.5 (C=O). ^77^Se NMR (76 MHz, CDCl_3_): δ 508.7. IR (KBr): *ν* 2931, 2853, 1608 (C=O), 1562 (C=C), 1442, 1347, 1243, 1225, 1128, 1012, 955, 788, 658, 631, 602, 538 cm^−1^. MS (EI), *m*/*z* (%): 556 (4) [M]^+^, 218 (7), 161 (9), 138 (47), 112 (14), 84 (100), 69 (17), 56 (12), 55 (14), 42 (9), 41 (23). Anal. calcd for C_16_H_24_N_2_O_2_Se (355.33): C 54.08, H 6.81, N 7.88, Se 22.22%. Found: C 53.81, H 6.82, N 7.91, Se 22.03%.

## 4. Conclusions

The efficient regio- and stereoselective synthesis of a novel class of organoselenium compounds, (*Z,Z*)-3,3′-selanediylbis(2-propenamides), based on the reaction of sodium selenide with 3-trimethylsilyl-2-propynamides was developed. Not a single representative of 3,3′-selanediylbis(2-propenamides) has yet been described in the literature. Studying their glutathione peroxidase-like properties by a model reaction showed that compounds **2g**,**i**,**d** exhibit high activity. It was found that the glutathione peroxidase-like activity of the obtained products varies significantly depending on the organic moieties in the amide group. Containing two lipophilic cyclohexyl substituents in the amide group compound **2g** is significantly superior to other products in activity. The second most active product is compound **2i** bearing the piperidine moieties in the amide function. Containing the morpholine and diphenyl moieties compounds **2f**,**h** also exhibit higher catalytic activity compared to diphenyl diselenide. 

## Supplementary Materials

The following are available online, the NMR spectra of the obtained compounds**.**
