# Supplementary material for: (Z,Z)-Selanediylbis(2-propenamides): Novel Class of Organoselenium Compounds with High Glutathione Peroxidase-Like Activity. Regio- and Stereoselective Reaction of Sodium Selenide with 3-Trimethylsilyl-2-propynamides
Source: Molecules. 2020 Dec 15;25(24):5940. doi: 10.3390/molecules25245940 (PMC7765452; doi:10.3390/molecules25245940)
Supplement: Supplementary file 1 [file molecules-25-05940-s001.pdf]

Supporting Information

(*Z*,*Z*)-Selanediylbis(2-propenamides): Novel Class of Organoselenium Compounds with High Glutathione Peroxidase-Like Activity. Regio- and Stereoselective Reaction of Sodium Selenide with 3-Trimethylsilyl-2-propynamides

Mikhail V. Andreev, Vladimir A. Potapov *, Maxim V. Musalov and Svetlana V. Amosova

A. E. Favorsky Irkutsk Institute of Chemistry, Siberian Division of The Russian Academy of Sciences, 1 Favorsky Str., Irkutsk 664033, Russian Federation; miand@irioch.irk.ru (M.V.A.); musalov_maxim@irioch.irk.ru (M.V.M.); amosova@irioch.irk.ru (S.V.A.)

* Correspondence: v.a.potapov@mail.ru

Table of contents

1. General information……………………………………………………..2

2. NMR spectra of products 2a-i…………………………………………..…3

1. General Information

The ^{1}H (400.1 MHz) and ^{13}C (100.6 MHz) NMR spectra were recorded on a Bruker DPX-400 and Bruker AV-400 spectrometers in $CDCl_3$ and d_6-DMSO 5-10% solutions. Chemical shifts (δ) in ppm are reported relative to the residual solvent peak of chloroform (7.27 for ^{1}H and 77.0 for ^{13}C) and DMSO (2.50 for ^{1}H and 39.6 for ^{13}C).

2. The NMR spectra of products 2a-i

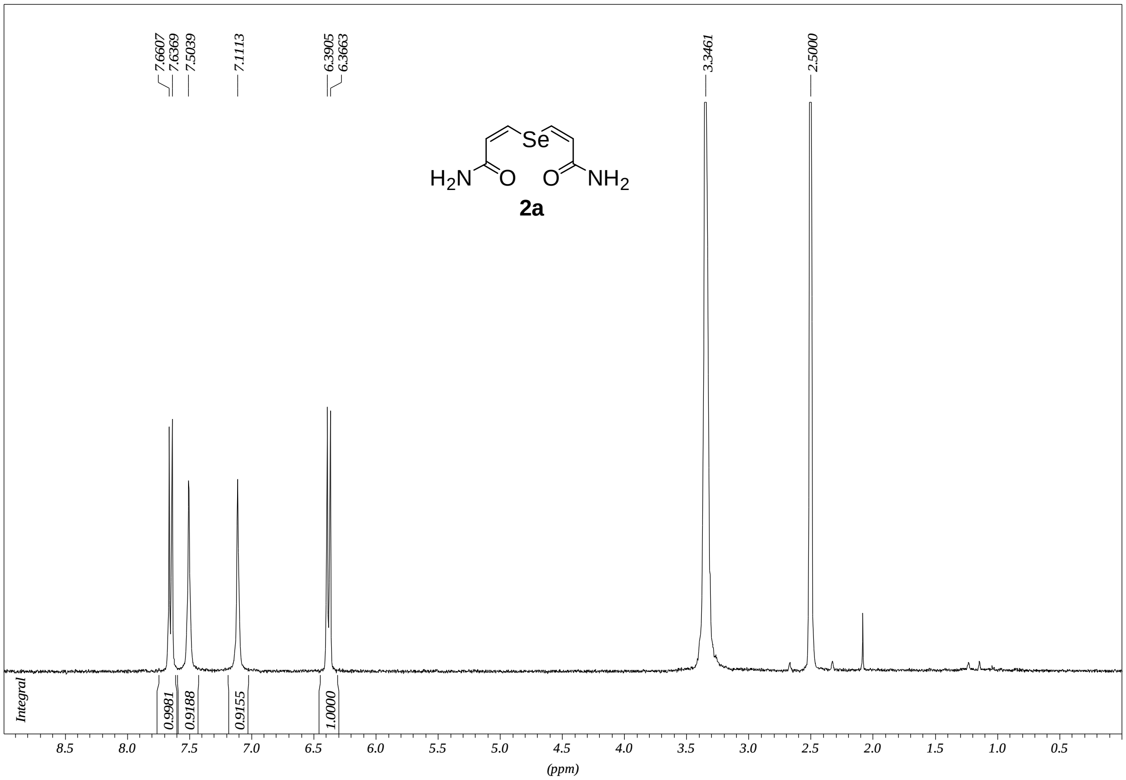

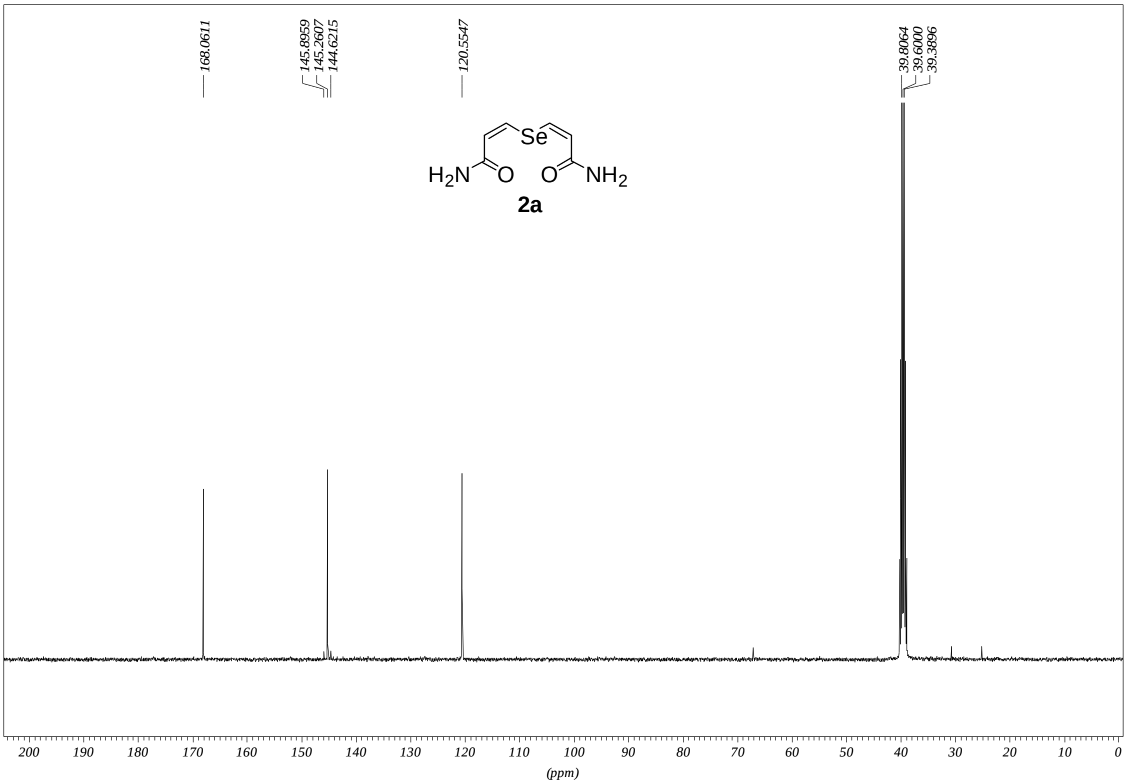

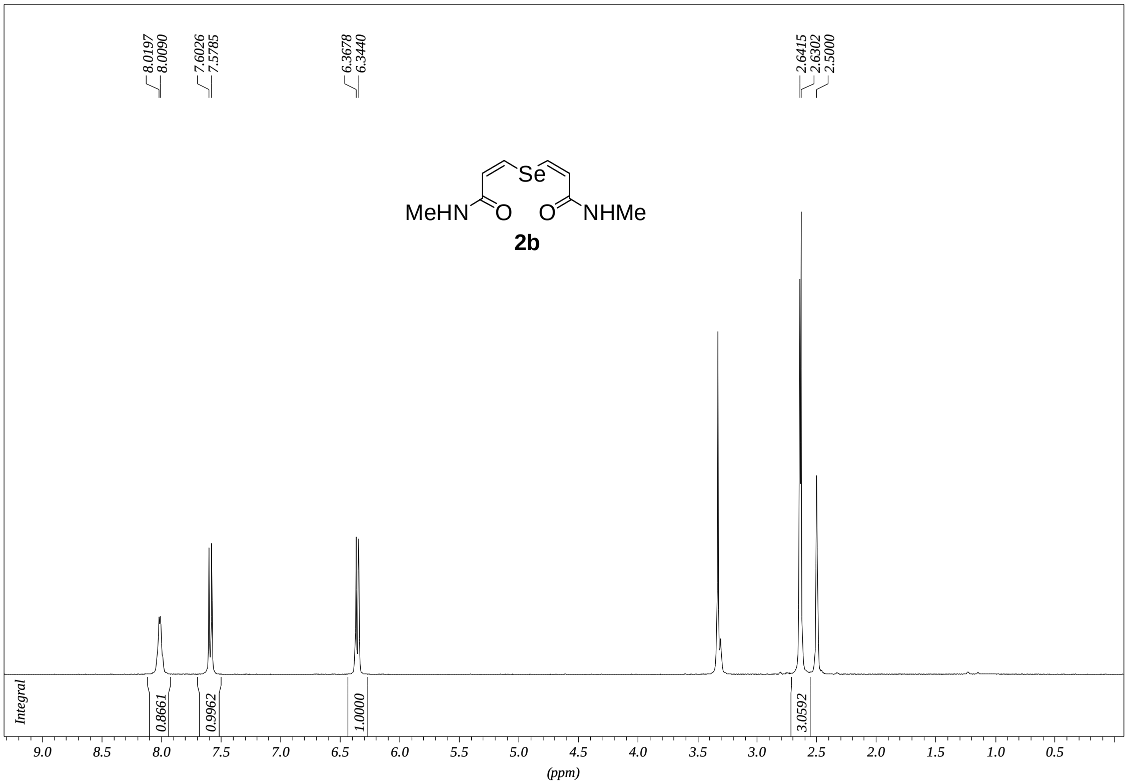
8.0197
8.0090
7.6026
7.5785
6.3678
6.3440
2.6415
2.6302
2.5000
Se
MeHN
O
O
NHMe
2b
Integral
0.8661
0.9962
1.0000
3.0592
9.0
8.5
8.0
7.5
7.0
6.5
6.0
5.5
5.0
4.5
4.0
3.5
3.0
2.5
2.0
1.5
1.0
0.5
(ppm)

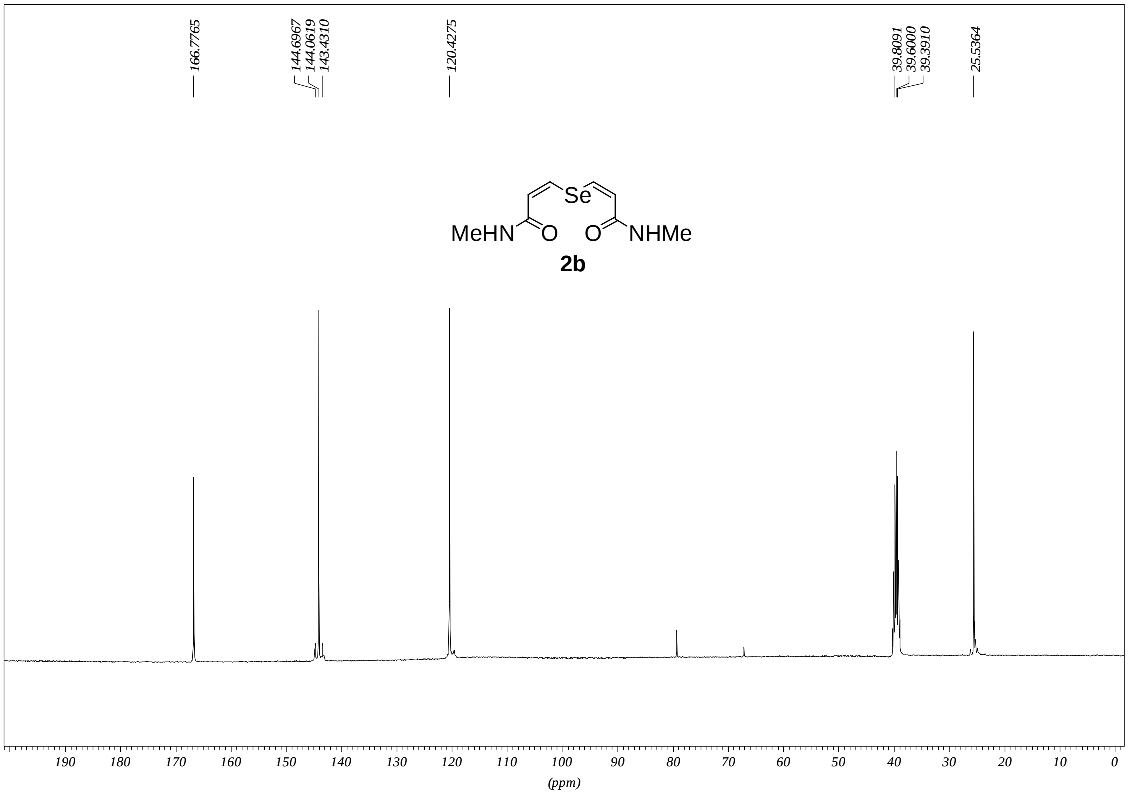
166.7765
144.6967
144.0619
143.4310
120.4275
39.8091
39.6000
39.3910
25.5364
Se
MeHN
O
O
NHMe
2b
190
180
170
160
150
140
130
120
110
100
90
80
70
60
50
40
30
20
10
0
(ppm)

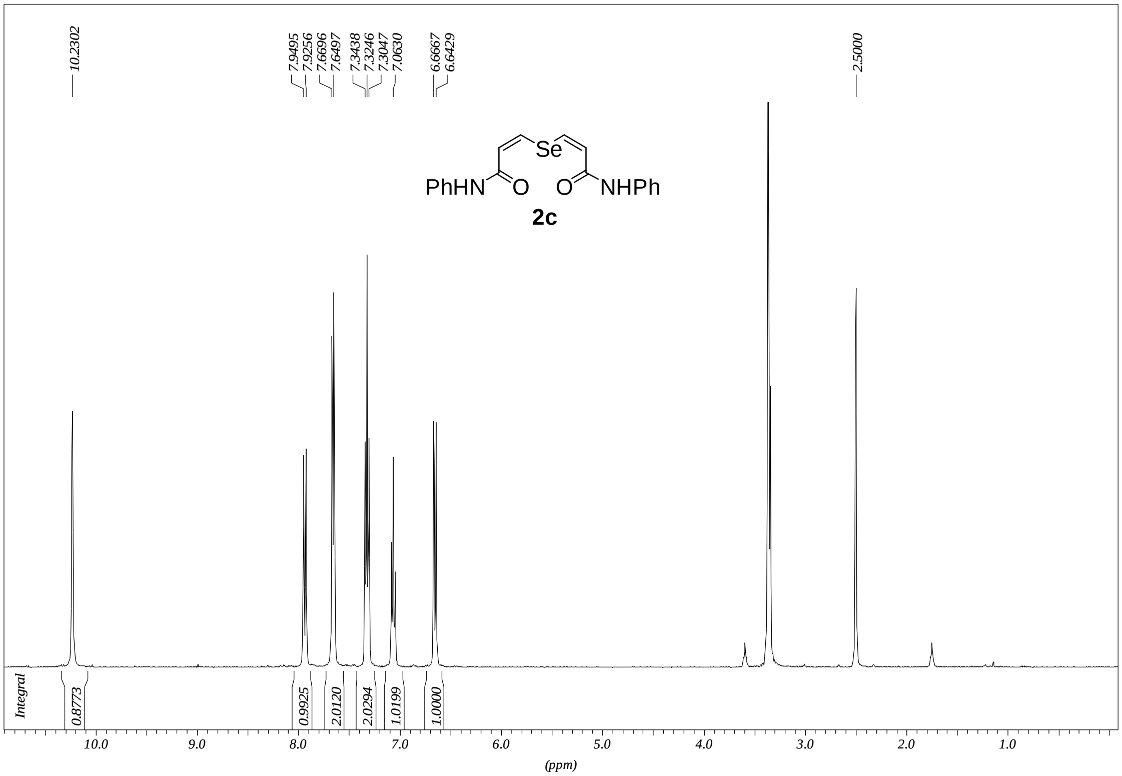
10.2302
7.9495
7.9256
7.6696
7.6497
7.3438
7.3246
7.3047
7.0630
6.6667
6.6429
2.5000
PhHN
Se
O
O
NHPh
2c
Integral
0.8773
0.9925
2.0120
2.0294
1.0199
1.0000
10.0
9.0
8.0
7.0
6.0
5.0
4.0
3.0
2.0
1.0
(ppm)

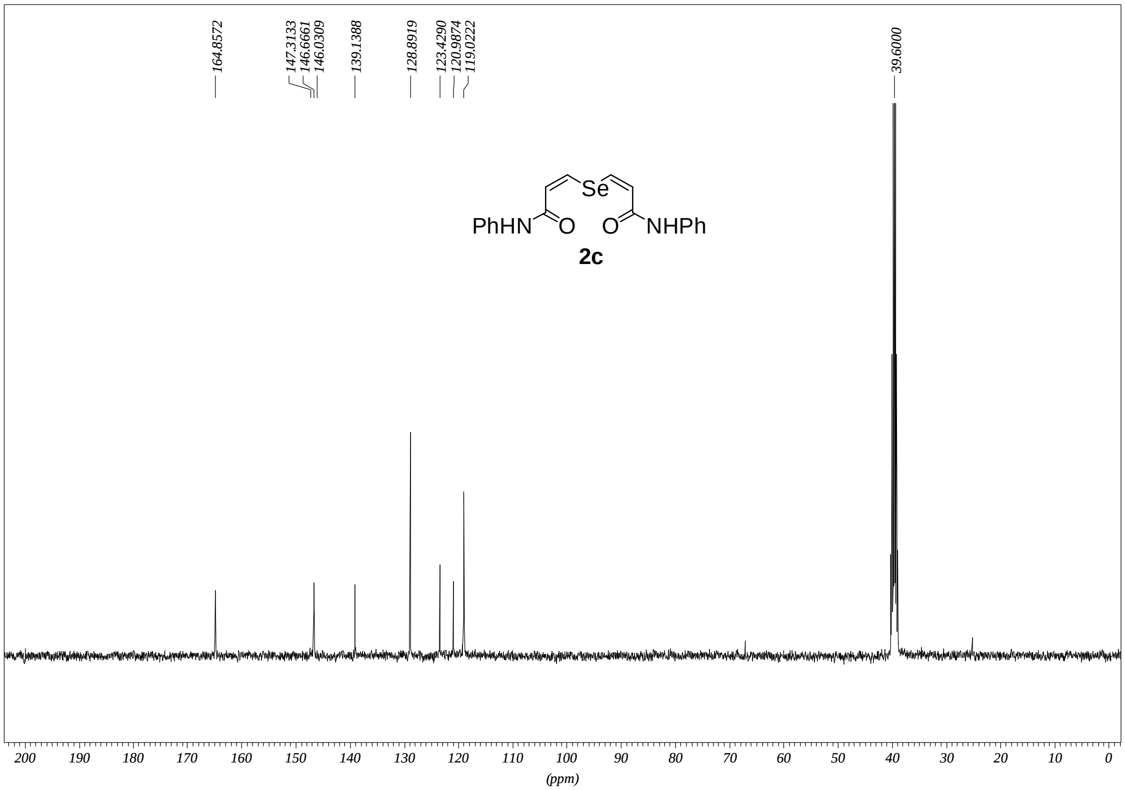
164.8572
147.3133
146.6661
146.0309
139.1388
128.8919
123.4290
120.9874
119.0222
39.6000
PhHN
Se
O
O
NHPh
2c
200
190
180
170
160
150
140
130
120
110
100
90
80
70
60
50
40
30
20
10
0
(ppm)

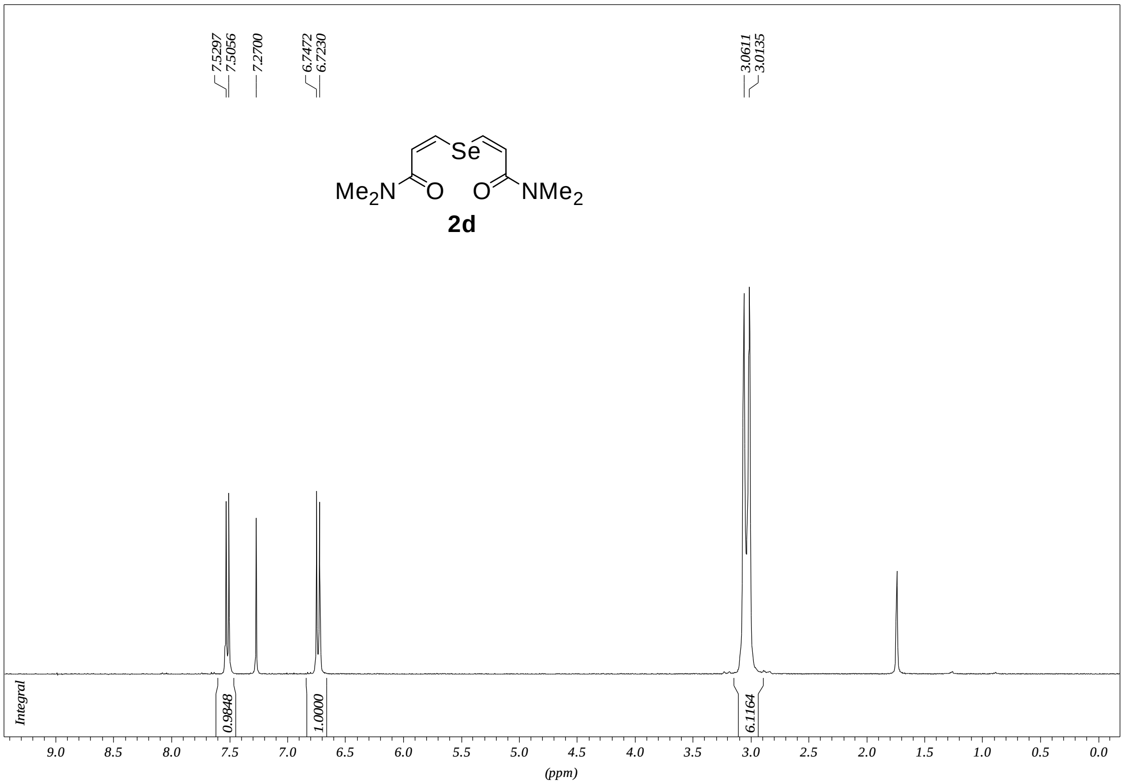
7.5297
7.5056
7.2700
6.7472
6.7230
3.0611
3.0135
Se
Me2N
O
O
NMe2
2d
Integral
0.9848
1.0000
6.1164
9.0 8.5 8.0 7.5 7.0 6.5 6.0 5.5 5.0 4.5 4.0 3.5 3.0 2.5 2.0 1.5 1.0 0.5 0.0
(ppm)

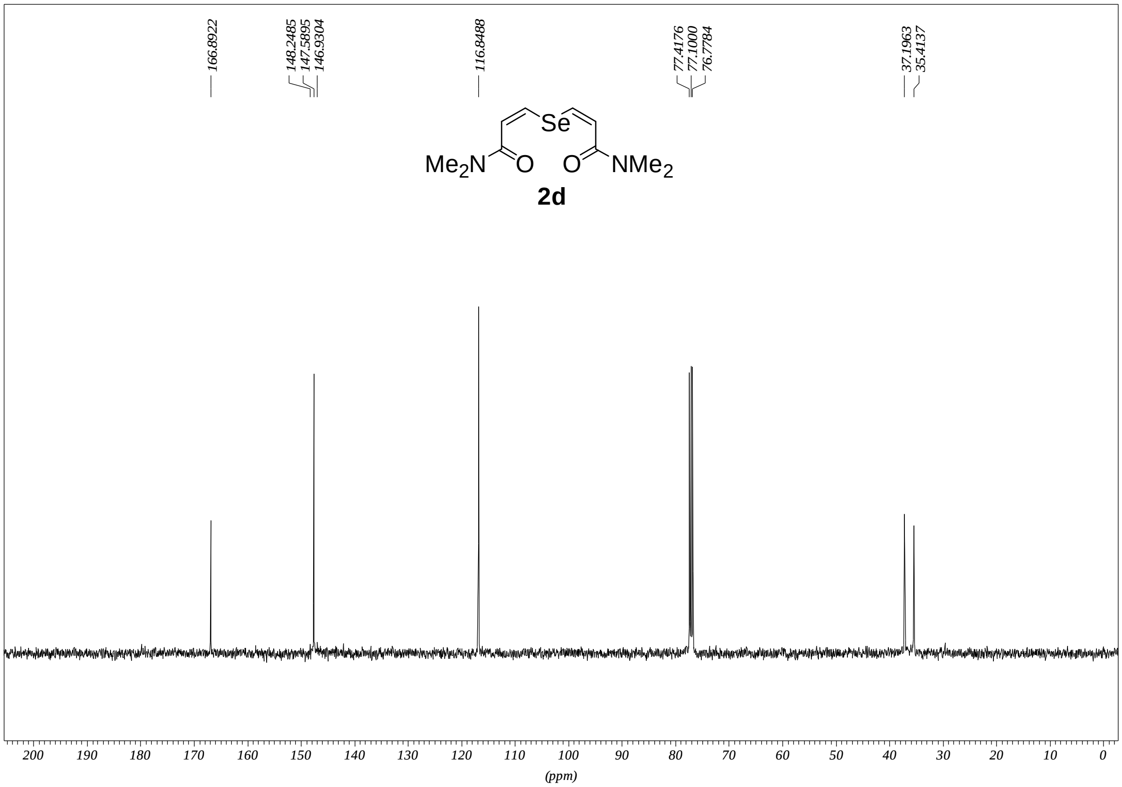
166.8922
148.2485
147.5895
146.9304
116.8488
77.4176
77.1000
76.7784
37.1963
35.4137
Se
Me2N
O
O
NMe2
2d
200 190 180 170 160 150 140 130 120 110 100 90 80 70 60 50 40 30 20 10 0
(ppm)

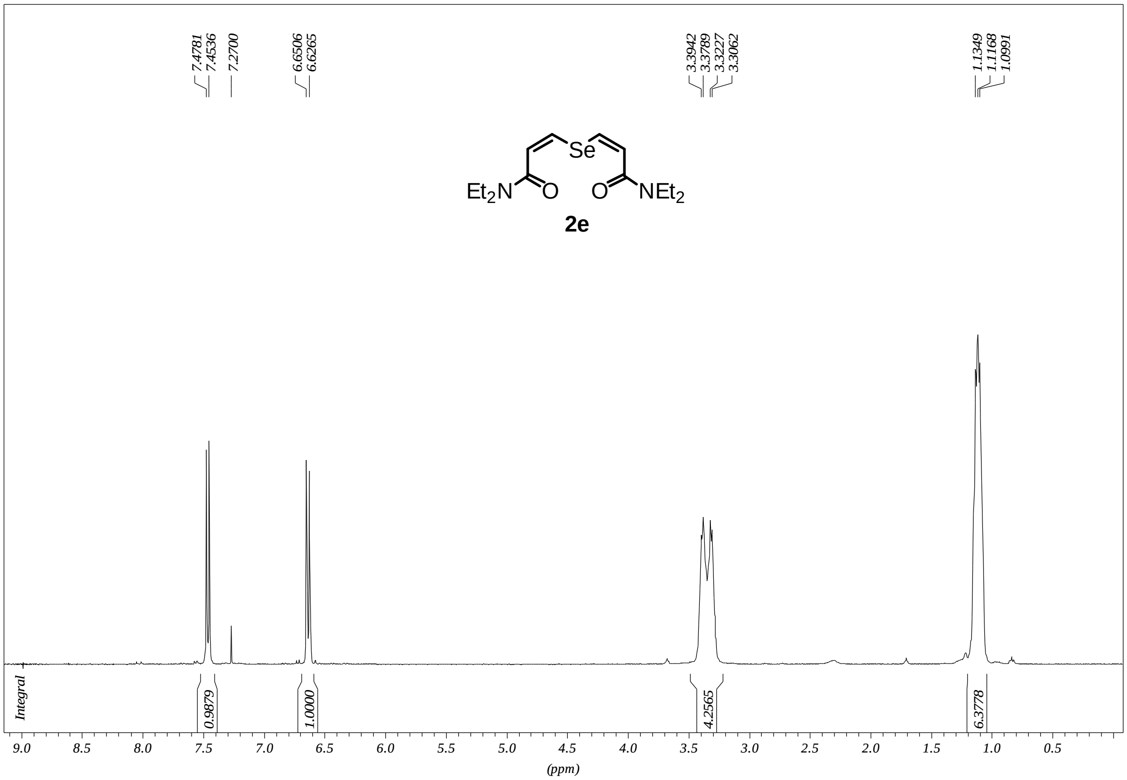
7.4781
7.4536
7.2700
6.6506
6.6265
3.3942
3.3789
3.3227
3.3062
1.1349
1.1168
1.0991
Se
Et2N
O
O
NEt2
2e
Integral
0.9879
1.0000
4.2565
6.3778
9.0
8.5
8.0
7.5
7.0
6.5
6.0
5.5
5.0
4.5
4.0
3.5
3.0
2.5
2.0
1.5
1.0
0.5
(ppm)

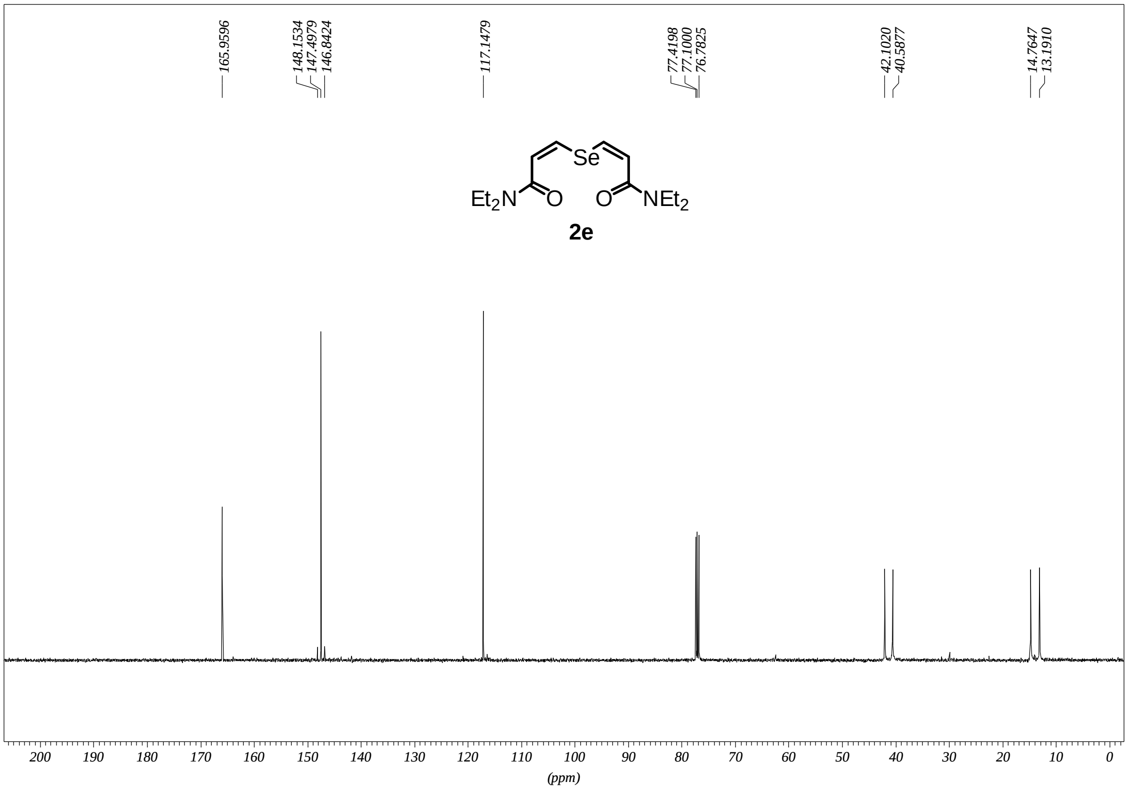
165.9596
148.1534
147.4979
146.8424
117.1479
77.4198
77.1000
76.7825
42.1020
40.5877
14.7647
13.1910
Se
Et2N
O
O
NEt2
2e
200
190
180
170
160
150
140
130
120
110
100
90
80
70
60
50
40
30
20
10
0
(ppm)

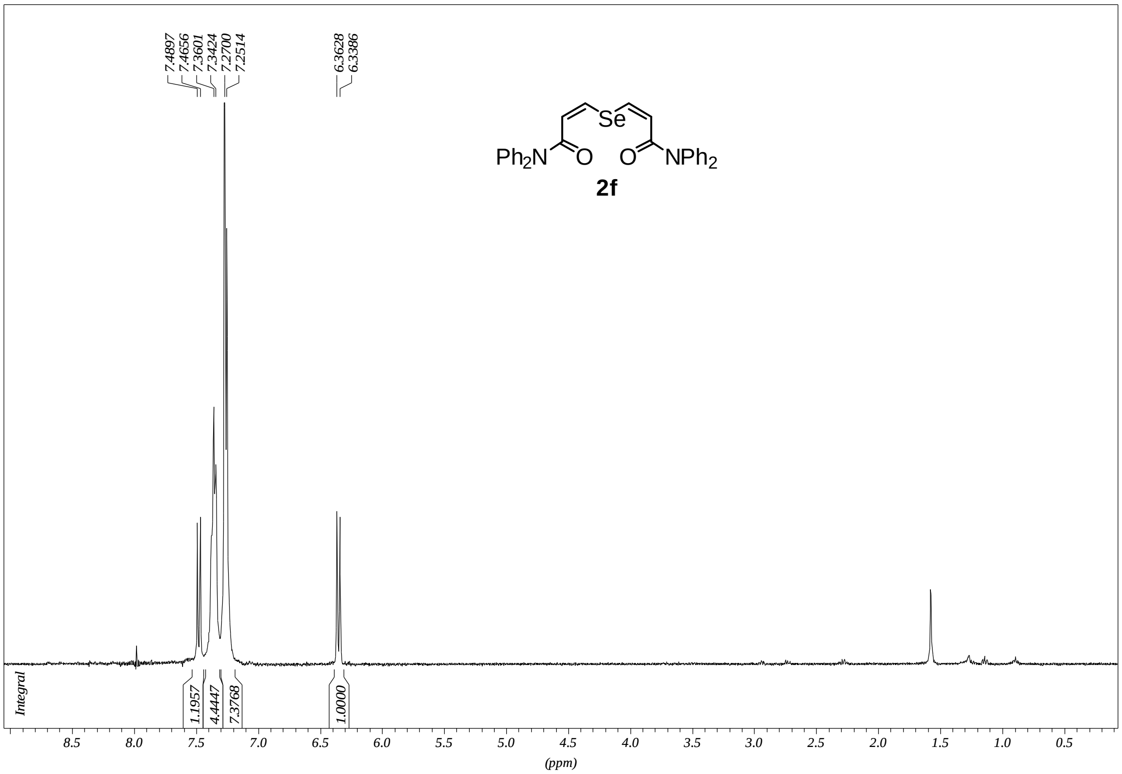
7.4897
7.4656
7.3601
7.3424
7.2700
7.2514
6.3628
6.3386
Se
Ph2N
O
O
NPh2
2f
Integral
1.1957
4.4447
7.3768
1.0000
8.5
8.0
7.5
7.0
6.5
6.0
5.5
5.0
4.5
4.0
3.5
3.0
2.5
2.0
1.5
1.0
0.5
(ppm)

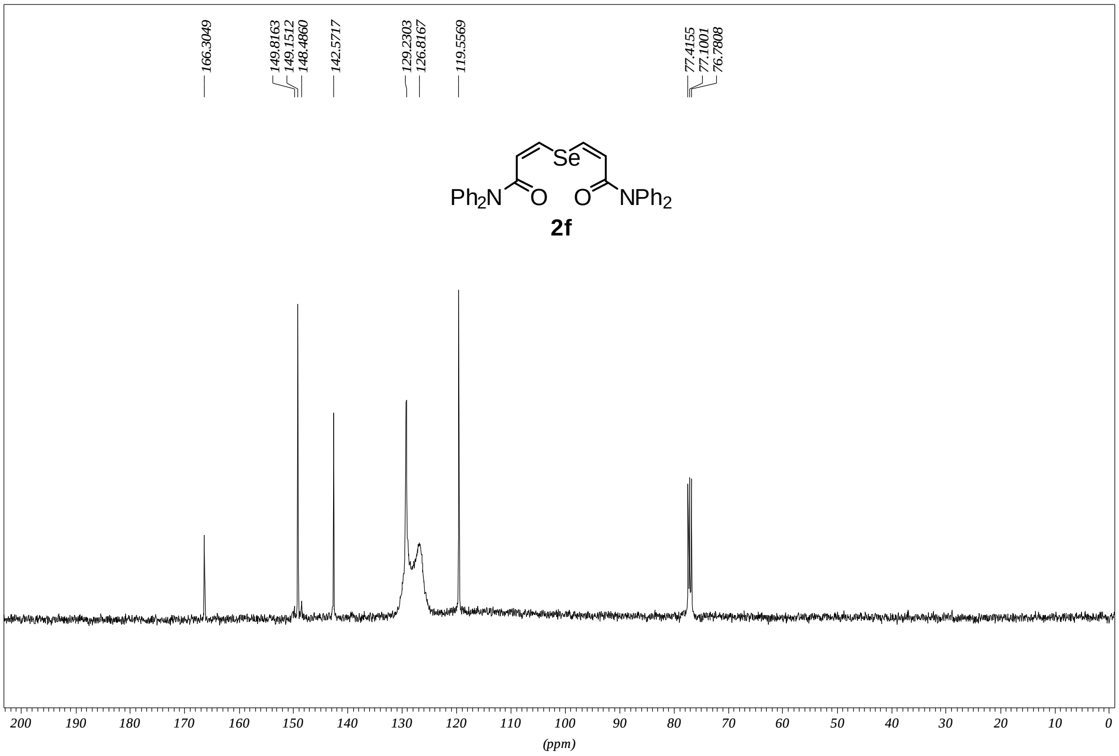
166.3049
149.8163
149.1512
148.4860
142.5717
129.2303
126.8167
119.5569
77.4155
77.1001
76.7808
Se
Ph2N
O
O
NPh2
2f
200
190
180
170
160
150
140
130
120
110
100
90
80
70
60
50
40
30
20
10
0
(ppm)

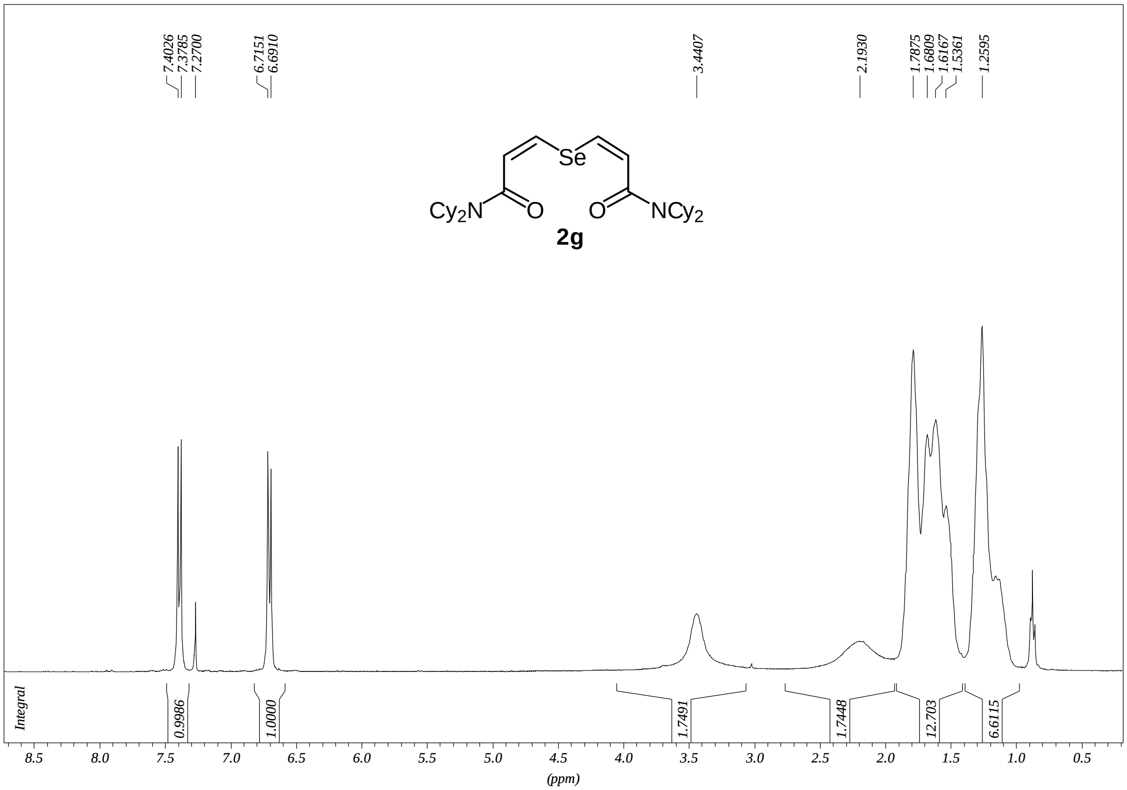
7.4026
7.3785
7.2700
6.7151
6.6910
3.4407
2.1930
1.7875
1.6809
1.6167
1.5361
1.2595
Se
Cy2N
O
O
NCy2
2g
Integral
0.9986
1.0000
1.7491
1.7448
12.703
6.6115
8.5 8.0 7.5 7.0 6.5 6.0 5.5 5.0 4.5 4.0 3.5 3.0 2.5 2.0 1.5 1.0 0.5
(ppm)

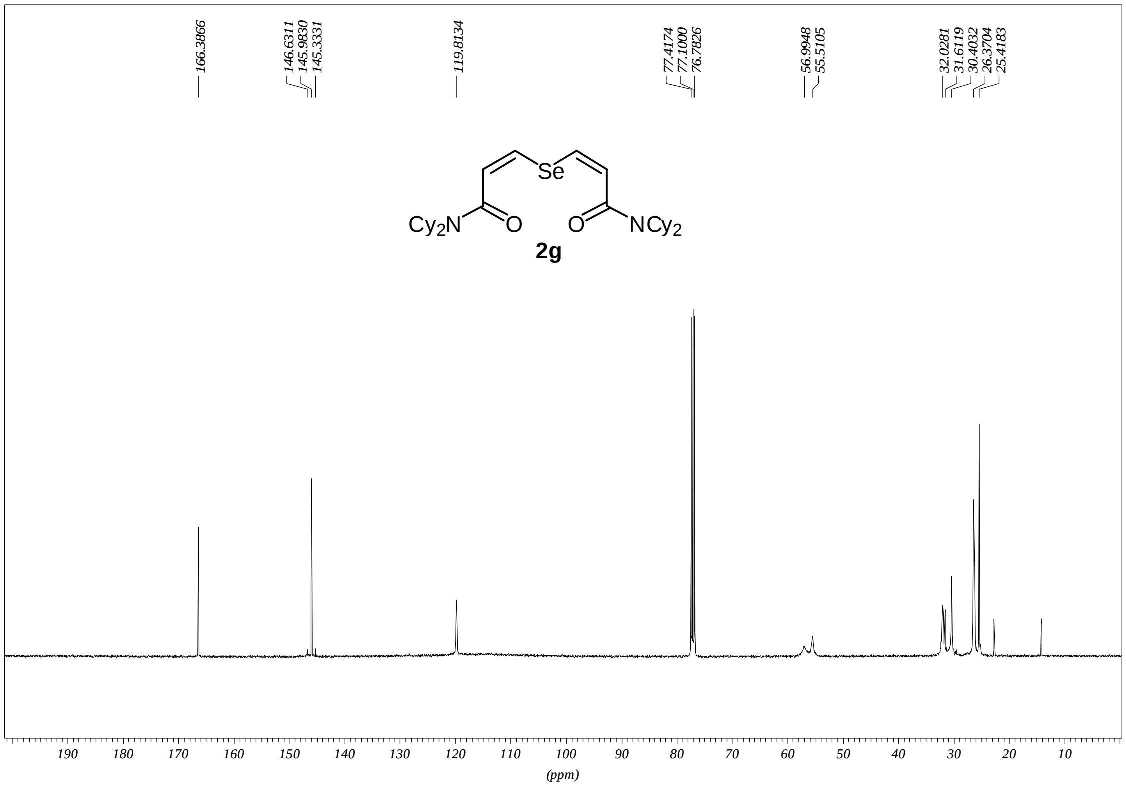
166.3866
146.6311
145.9830
145.3331
119.8134
77.4174
77.1000
76.7826
56.9948
55.5105
32.0281
31.6119
30.4032
26.3704
25.4183
Se
Cy2N
O
O
NCy2
2g
190 180 170 160 150 140 130 120 110 100 90 80 70 60 50 40 30 20 10
(ppm)

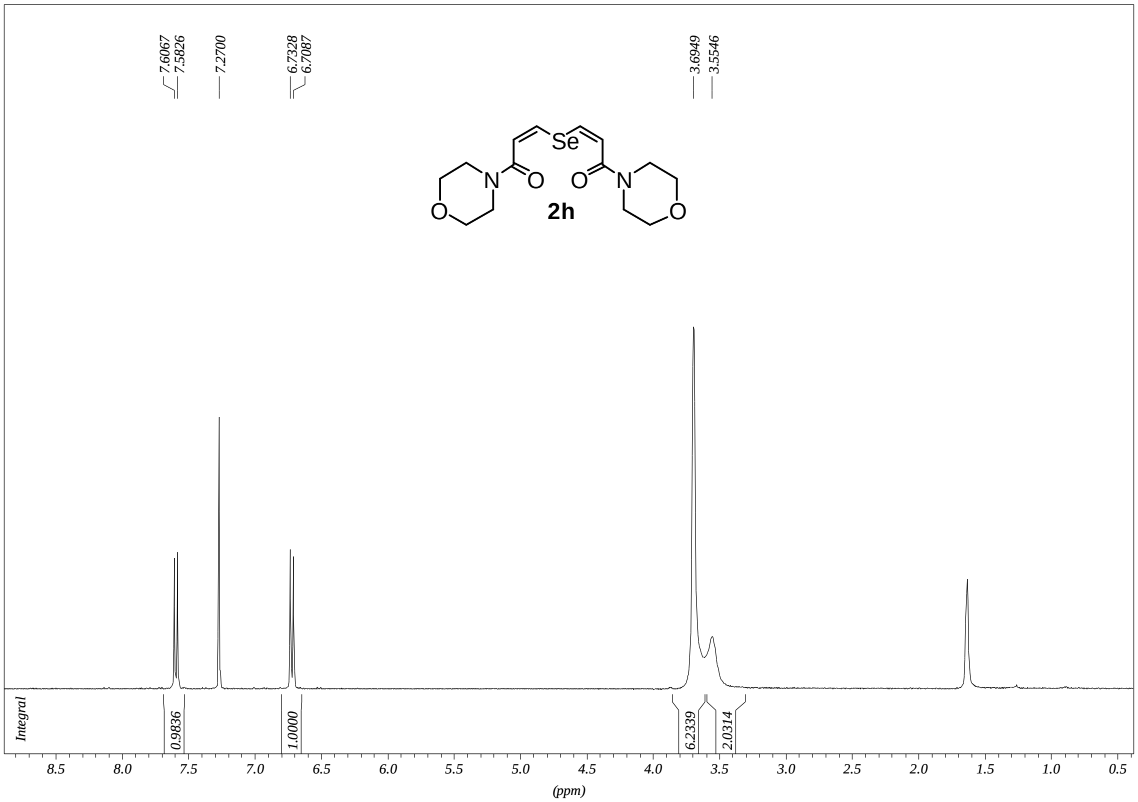
7.6067
7.5826
7.2700
6.7328
6.7087
3.6949
3.5546
Se
N
O
O
N
O
O
2h
Integral
0.9836
1.0000
6.2339
2.0314
8.5
8.0
7.5
7.0
6.5
6.0
5.5
5.0
4.5
4.0
3.5
3.0
2.5
2.0
1.5
1.0
0.5
(ppm)

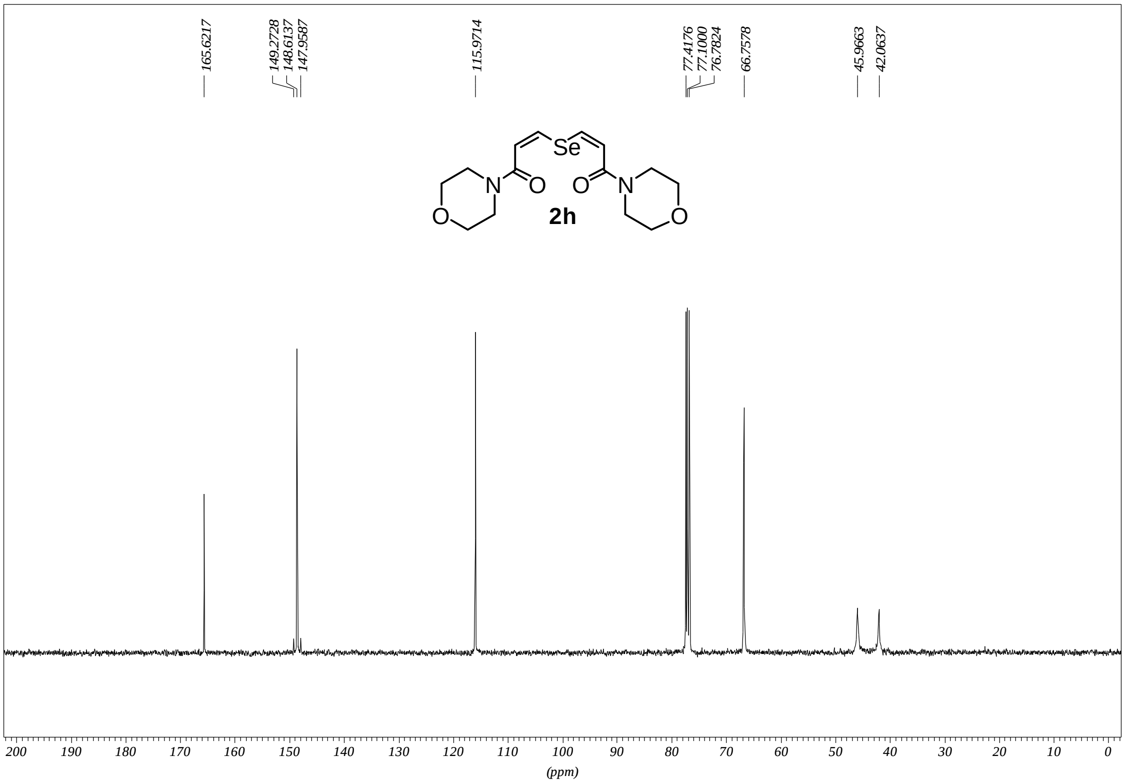
165.6217
149.2728
148.6137
147.9587
115.9714
77.4176
77.1000
76.7824
66.7578
45.9663
42.0637
Se
N
O
O
N
O
O
2h
200
190
180
170
160
150
140
130
120
110
100
90
80
70
60
50
40
30
20
10
0
(ppm)

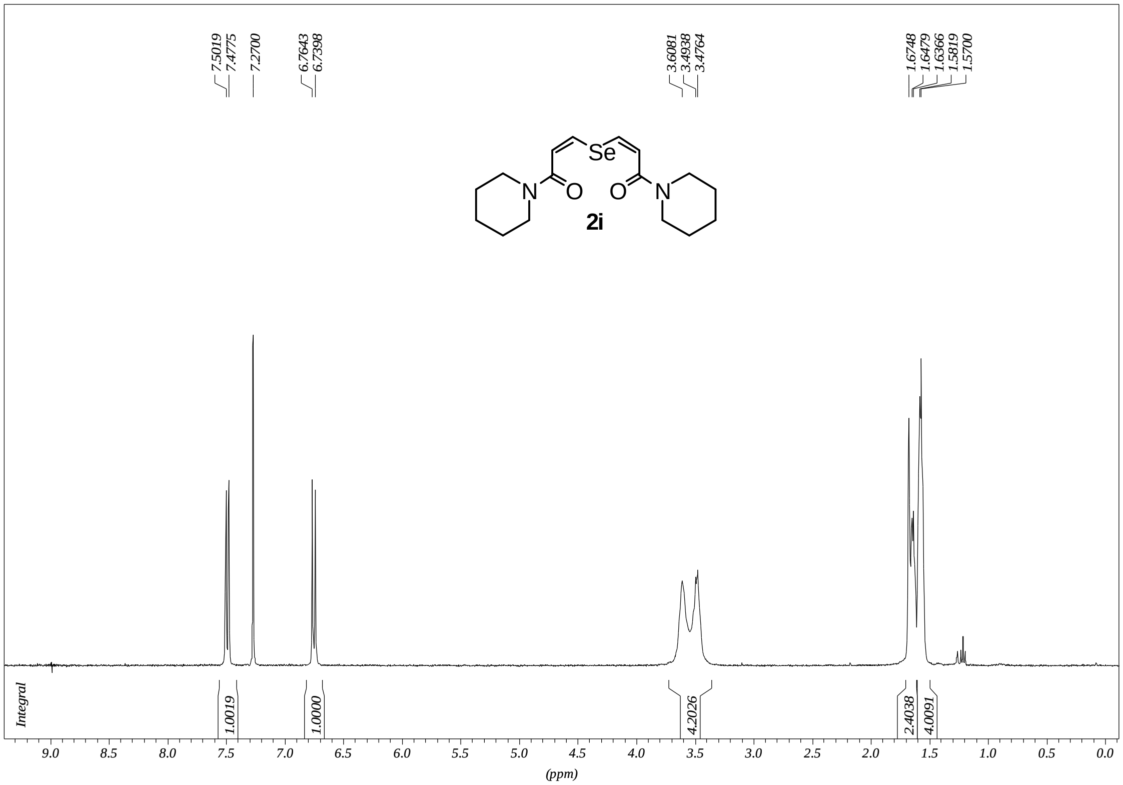
7.5019
7.4775
7.2700
6.7643
6.7398
3.6081
3.4938
3.4764
1.6748
1.6479
1.6366
1.5819
1.5700
Se
N
O
O
N
2i
Integral
1.0019
1.0000
4.2026
2.4038
4.0091
9.0 8.5 8.0 7.5 7.0 6.5 6.0 5.5 5.0 4.5 4.0 3.5 3.0 2.5 2.0 1.5 1.0 0.5 0.0
(ppm)

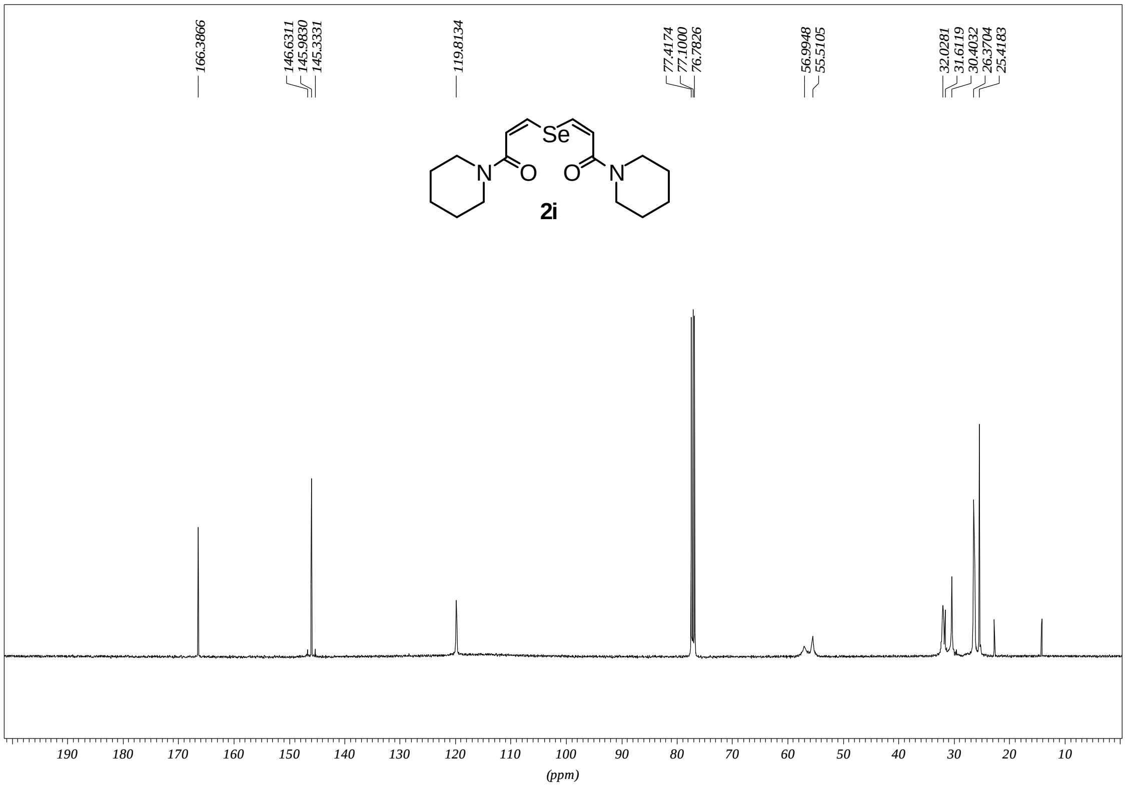
166.3866
146.6311
145.9830
145.3331
119.8134
77.4174
77.1000
76.7826
56.9948
55.5105
32.0281
31.6119
30.4032
26.3704
25.4183
Se
N
O
O
N
2i
190 180 170 160 150 140 130 120 110 100 90 80 70 60 50 40 30 20 10
(ppm)

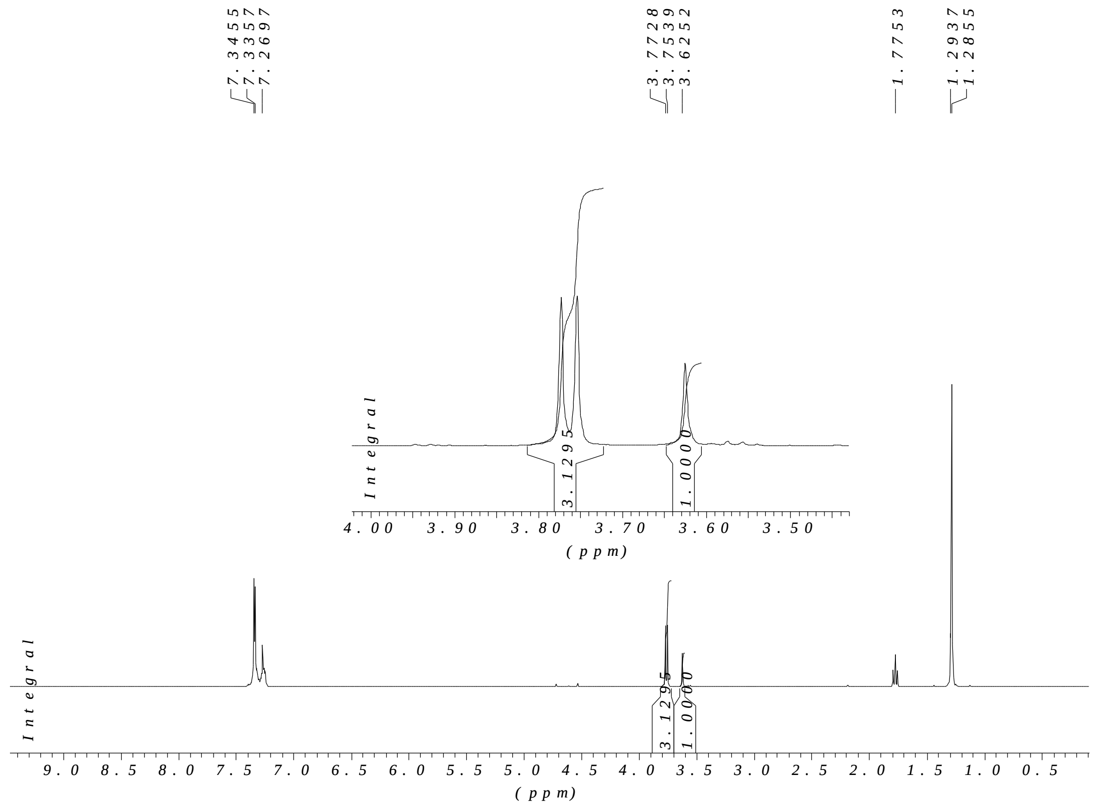

Typical experiment on studying the glutathione peroxidase-like activity of the obtained compounds (0.5% mol) by ^{1}H NMR monitoring (TBHP, BnSH, 0.1 mmol, deuterochloroform, the 24% conversion of phenylmethanethiol)
